# Evaluating exon skipping in the central nervous system in Duchenne muscular dystrophy using spatial transcriptomics

**DOI:** 10.1016/j.isci.2026.116906

**Published:** 2026-07-23

**Authors:** Qirong Mao, Alireza Ahmadi, Sharon de Vries, Laura G.M. Heezen, Ophélie Vacca, Mathilde Doisy, Annemieke Aartsma-Rus, Maaike van Putten, Aurélie Goyenvalle, Ahmed Mahfouz, Pietro Spitali

**Affiliations:** 1 Department of Human Genetics, Leiden University Medical Center, Leiden, the Netherlands; 2 Université Paris-Saclay, UVSQ, Inserm, END-ICAP, Versailles, France; 3 Duchenne Center Netherlands, the Netherlands; 4 Delft Bioinformatics Lab, Delft University of Technology, Delft, the Netherlands

**Keywords:** Duchenne muscular dystrophy, central nervous system, dystrophin isoforms, exon-skipping therapy, spatial transcriptomics

## Abstract

Duchenne muscular dystrophy (DMD) causes progressive muscle degeneration due to dystrophin deficiency. Dystrophin is also expressed in the brain during development and postnatally, yet a characterization of dystrophin isoform expression across brain cells and regions is lacking, limiting our understanding of the cognitive impairment affecting one-third of the patients and hampering the development of dystrophin-restoring drugs in the central nervous system (CNS). Here, we applied spatial transcriptomics to map *Dmd* isoforms across mouse brain regions and cell types. *Mdx52* mice received exon 51-skipping therapies restoring the Dp427-sized isoform at the transcript and protein levels. We observed distinct spatial patterns: full-length isoforms localized to deeper cortical layers and CA1, while shorter isoforms were enriched in cortical layer 1 and dentate gyrus. We present evidence of isoform restoration, immune activation following treatment, and a framework to evaluate exon-skipping therapies in the CNS using spatial transcriptomics.

## Highlights

• Spatial transcriptomics maps *Dmd* isoforms across cell types and brain regions
• Full-length and shorter *Dmd* isoforms show distinct regional and cellular patterns
• Spatial transcriptomics enables studying antisense-mediated exon skipping in the CNS
• Antisense treatment increases microglia in the mouse brain, independent of chemistry

## Graphical abstract

**Figure:**
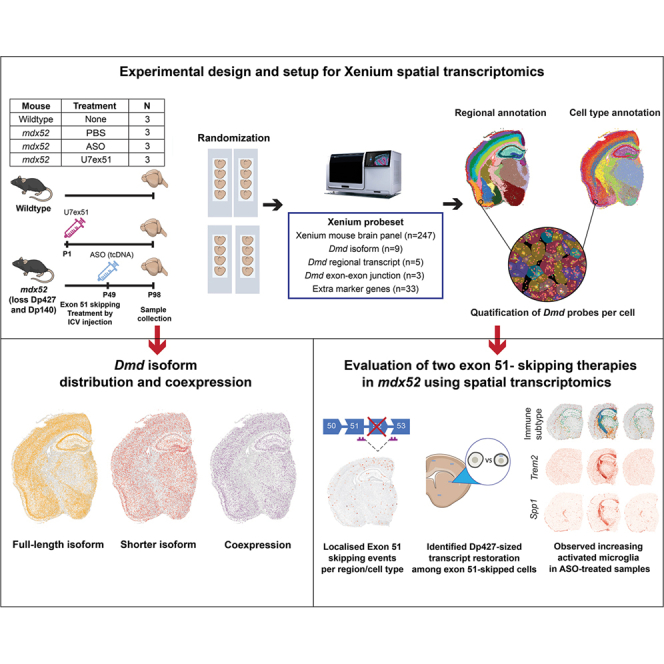

## Teaser

Therapeutics; Genetics; Neuroscience; Transcriptomics

## Introduction

Duchenne muscular dystrophy (DMD) is an X-linked neuromuscular disorder caused by mutations in the *DMD* gene, which encodes dystrophin. Nonsense and frameshift mutations in the *DMD* gene cause the absence of functional dystrophin in DMD. Along with the progressive loss of muscle tissue and function, DMD is also associated with cognitive disorders. Nearly one-third of patients with DMD show mild to severe intellectual disability, with a full-scale intelligence quotient (FSIQ) lower than 70 and an overall mean FSIQ decreasing by one standard deviation compared to normal populations.^1^^,^^2^^,^^3^ Additionally, a higher prevalence of neurodevelopmental disorders has been observed among patients with DMD, such as emotional dysregulation, epilepsy, attention-deficit/hyperactivity disorder (ADHD), autism spectrum disorder (ASD), and obsessive-compulsive disorder (OCD).^4^^,^^5^^,^^6^^,^^7^^,^^8^^,^^9^ Recent studies have also shown that patients with DMD frequently exhibit academic challenges, such as reading difficulties and deficits in mathematical reasoning abilities.^10^^,^^11^^,^^12^ Over the past decades, advances in care strategies have significantly improved disease management. However, relatively little attention has been given to investigating brain comorbidities in DMD. These challenges continue to negatively impact the quality of life of patients with DMD, underscoring the urgent need for further research and targeted interventions.

The *DMD* gene has seven tissue-specific promoters, enabling the production of various dystrophin protein (Dp) isoforms named according to their molecular size. Three full-length 427 kDa isoforms are encoded by three separate promoters located upstream of the first exon: Dp427c is present in neurons of the cerebral cortex and cerebellum, Dp427m is mainly expressed in skeletal, cardiac and smooth muscle, and Dp427p is expressed in the Purkinje cells in the cerebellum.^13^^,^^14^ In addition, shorter dystrophin isoforms (Dp260, Dp140, Dp116, Dp71, and Dp40) are transcribed from downstream promoters located in the introns of the *DMD* gene. Dp260 is predominantly localized to the retina, and Dp116 is primarily expressed in the peripheral nerve.^15^^,^^16^ Dp140, Dp71, and Dp40 are expressed in the central nervous system (CNS) as well as in other tissues. Dp140 is also expressed in the kidney, whereas Dp71 and Dp40 are broadly expressed across multiple tissues and are highly expressed in the CNS.^13^^,^^17^

Brain comorbidities have been linked to the mutation sites along the gene, with severity and frequency of phenotypes being higher for patients carrying pathogenic variants located in the distal part of the *DMD* gene, leading to a cumulative loss of dystrophin isoforms.^18^^,^^19^ A meta-analysis has shown that patients with DMD lacking Dp427 have lower average IQ scores, with progressive reduction when Dp140 and Dp71 are also absent.^20^ Distal mutations affecting Dp140/Dp71 are associated with a higher prevalence of ASD and ADHD diagnoses, as well as emotional and behavioral problems, although variability between patients remains substantial.^21^^,^^22^^,^^23^ In addition, patients with DMD missing only Dp427 show better motor performance than those who also missing Dp140 and/or Dp71.^24^^,^^25^

Over the past decades, dystrophin-restoring therapies such as antisense oligonucleotide (ASO)-mediated exon skipping have shown promise for treating skeletal muscles pathology in DMD.^26^ ASOs are small synthetic molecules that interfere with pre-mRNA splicing, causing the exclusion of specific exon(s) from the mature mRNA. Such an approach has been shown to restore the open reading frame and to allow the synthesis of truncated but partially functional dystrophins.^27^ Clinical trials have demonstrated only minimal dystrophin restoration in the skeletal muscle of patients with DMD following ASO treatment, leading to the accelerated approval by the U.S. Food and Drug Administration (FDA) of four ASOs, and by Japan’s Ministry of Health, Labour and Welfare (MHLW) of one ASO.^28^^,^^29^^,^^30^^,^^31^

In addition to ASOs, antisense sequences can also be introduced into cells through viral delivery of the antisense sequence as part of a uridine-rich 7 small nuclear RNA (U7snRNA) cassette. U7snRNA is part of the small nuclear ribonucleoprotein and participates in 3′ end processing of histone pre-mRNA.^32^ With the modification of the binding site for Sm/Lsm protein, U7snRNA can mediate splicing and induce skipping/inclusion of target exons.^33^ Delivery of U7snRNA with an antisense sequence has been explored using adeno-associated viral vectors (AAV), showing therapeutic potential in DMD mouse and dog models, and has been used in a small-scale open-label clinical trial.^34^^,^^35^^,^^36^

Despite therapeutic advances in dystrophin restoration targeting muscle in patients with DMD, the brain is left untreated as these ASOs show limited penetration across the blood-brain barrier.^37^ However, recent preclinical studies indicate this limitation may be overcome by chemical oligonucleotide modifications, including tricyclo-DNA (tcDNA) ASOs.^38^ Nevertheless, efforts to develop exon-skipping treatments addressing brain comorbidities in DMD remain scarce and in their infancy. One of the barriers is the limited knowledge of dystrophin isoforms’ function and localization across brain regions and cell types in the brain.^38^

Preliminary studies explored the potential of ASOs to restore dystrophin expression and alleviate brain-related symptoms in *mdx52* mice (which carry a deletion of exon 52 and consequently lack the expression of Dp427 and Dp140).^38^ A single intracerebroventricular (ICV) administration of ASOs targeting *Dmd* exon 51 partially restored 5%–15% of Dp427 protein expression in multiple brain regions, including the hippocampus, cerebellum, and cortex. Behaviorally, this treatment significantly reduced anxiety and unconditioned fear but only partially improved fear memory, suggesting that the modest behavioral benefits could result from the limited levels of Dp427 restoration and/or the relatively late (adult) timing of treatment. These findings raised the key question of whether higher dystrophin restoration or earlier intervention might lead to stronger functional recovery. This motivated us to explore alternative delivery strategies. In the present study, we therefore evaluated ICV administration of AAV-U7 in neonatal *mdx52* mice, directly comparing its effects with the previously described ICV administration of ASO in adult animals, which was associated with functional benefit.^38^

In this study, we applied Xenium spatial transcriptomics to map *Dmd* isoform localization and exon-skipping events in the mouse brain. This approach allowed us to describe the spatial distribution of *Dmd* isoforms in the brain at the cellular level. We further evaluated the restoration of Dp427-sized transcript and protein levels in the treated brain to see whether spatial transcriptomics could be useful to investigate further exon-skipping approaches in the CNS.

## Results

### Quality assessment and annotation of spatial transcriptomics data

**Figure 1:**
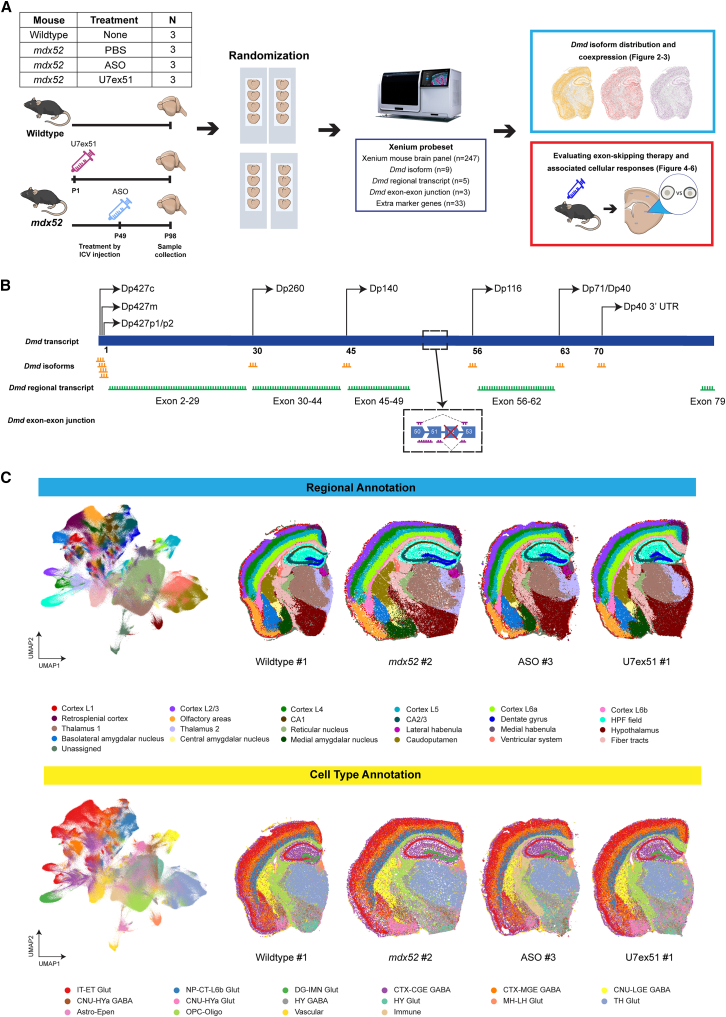
Spatial profiling of brain structure and cellular composition in DMD mouse model and wildtype control (A) Overview of the experimental design and downstream data analysis workflow. (B) Schematic representation of the custom Xenium panel design, including probes targeting isoform promoters or untranslated regions (probes shown in yellow), *Dmd* regional transcripts (probes shown in green), and exon-exon junctions (probes shown in purple). (C) Uniform Manifold Approximation and Projection (UMAP) plots for all samples, with spatial plots showing (Top) regional and (Bottom) cell type annotation of selected replicates per group. (Astro, astrocyte; CGE, caudal ganglionic eminence; CNU, cerebral nuclei; CT, corticothalamic; CTX, cerebral cortex; Epen, ependymal; ET, extratelencephalic; GABA, GABAergic; Glut, glutamatergic; HPF, hippocampal formation; HY, hypothalamus; HYa, anterior hypothalamic; IMN, immature neurons; IT, intratelencephalic; L6b, layer 6b; LGE, lateral ganglionic eminence; LH, lateral habenula; MGE, medial ganglionic eminence; MH, medial habenula; NP, near-projecting; Oligo, oligodendrocytes; OPC, oligodendrocyte precursor cells; TH, thalamus, nomenclature follows Yao et al.^39^).

We performed Xenium *in situ* sequencing (10x Genomics) on hemi-coronal brain sections from 12 individual mice across four groups: wildtype (*n* = 3), *mdx52* mice treated with PBS (*n* = 3), *mdx52* mice treated with either ASO (tcDNA) (*n* = 3) or AAV-U7snRNA targeting *Dmd* exon 51 skipping (U7ex51) (*n* = 3) (Materials and Methods). All samples were collected at 14 weeks of age (Figure 1A).

Here, we employed the pre-designed Xenium mouse brain panel with 247 genes, in addition to a custom add-on panel of 50 genes. To investigate the spatial distribution of *Dmd* isoforms across brain regions and cell types, we included custom probes to detect *Dmd* isoforms and *Dmd* transcript regions (proximal: Exon 2–29 and Exon 30–44, middle: Exon 45-49 and Exon 56–62, and distal: Exon 79). We also included *Dmd* exon-exon junction probes to evaluate the efficacy of exon 51-skipping therapy (Figure 1B; Table S1).

Overall, we detected 949,583 cells with an average of 91 (91.34 ± 31.54) genes per cell across the 12 samples (Figures S1–S3). Segmented cells were annotated at the cell type level by transferring labels from the Allen Brain Atlas P56 mouse brain single-cell RNA-seq reference using marker genes overlapped with our panel.^39^ We also annotated cells in different brain regions based on unsupervised clustering guided by anatomical information from the Allen Brain Atlas (Figures S4 and S5). Ultimately, we identified 24 brain regions and 16 distinct cell types (Figures 1C and S6–S11).

### Spatial localization of *Dmd* isoforms in the wildtype brain

**Figure 2:**
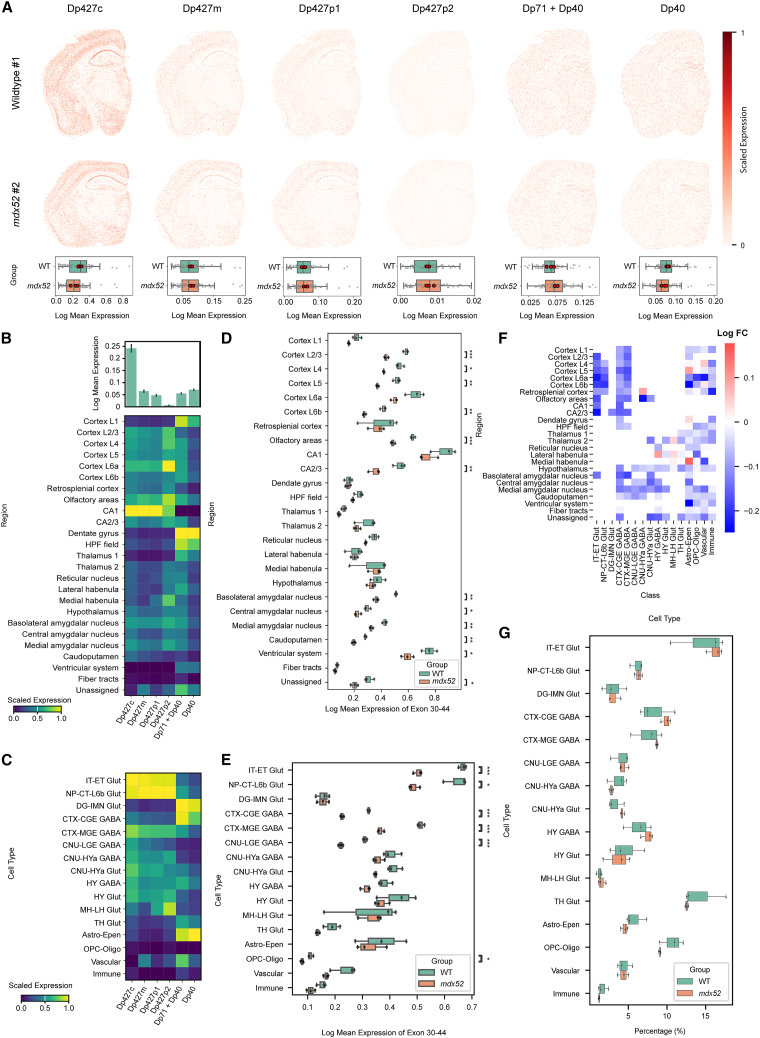
Distinct distribution patterns of *Dmd* isoforms and the differential expression of the full-length *Dmd* isoform across brain regions and cell types between wildtype and *mdx52* mice (A) (Top) Spatial distribution of *Dmd* isoforms in WT and *mdx52* mouse brains colored by the scaled expression of each *Dmd* isoform. (Bottom) Boxplot with overlaid dot plots showing the log-transformed mean expression of each *Dmd* isoform across samples and brain regions (black) and the log-transformed mean expression per mouse in WT and *mdx52* groups (red). Statistical analysis was performed using Welch’s *t* test with Benjamini-Hochberg (BH) correction at the sample-level comparison. (B and C) Expression of *Dmd* isoforms in WT mice and their log-normalized expression across brain regions and cell types. Results are shown in mean ± Standard Error of the Mean (SEM). (D and E) Expression of exon 30–44 across brain regions and cell types in WT and *mdx52* mice. Statistical analysis by Welch’s *t* test with BH correction. ∗*p* < 0.05, ∗∗*p* < 0.01, and ∗∗∗*p* < 0.001. (F) Log fold changes in exon 30–44 expression across brain regions and cell types between WT and *mdx52* mice. (G) Cell type proportions in brain samples of WT and *mdx52* mice. Proportions were compared between groups using Scanpro with Benjamini-Hochberg correction (see STAR Methods).

We examined the spatial distribution of multiple *Dmd* isoforms across our samples using promoter-specific probes. Probes for the Dp260 and Dp140 promoters were excluded from the downstream analysis due to insufficient detection signal (Figure S12). Dp427c was the predominant isoform in the WT brain, while Dp427m, Dp427p1, the first exon shared by Dp71 and Dp40 (Dp71 + Dp40), and the 3′UTR of Dp40 (Dp40) had lower expression levels. Dp427p2 showed the lowest expression level across the brain (Figures 2A and 2B).

The expression of *Dmd* isoforms across brain regions revealed two common patterns: one for the full-length isoforms (Dp427c, Dp427m, Dp427p1) and the other for the shorter isoforms (Dp71 + Dp40, Dp40). The full-length isoforms were mainly expressed in the cortex from layer 2/3 to layer 6b, olfactory areas, CA1, CA2/3, and basolateral amygdalar nucleus, while the shorter isoforms were mainly expressed in cortical layer 1, the dentate gyrus, and the HPF field (Figure 2B). We also found a distinct pattern at the cellular level: The full-length isoforms showed high expression in IT-ET Glut, NP-CT-L6b Glut, CTX-MGE GABA, CNU-HYa GABA, and HY Glut, while the shorter isoforms were predominantly expressed in DG-IMN Glut, CTX-CGE GABA and Astro-Epen cells. Moreover, we observed that Dp427m showed higher expression levels in vascular cells compared to the other full-length isoforms (Figure 2C).

### *Mdx52* mice show altered full-length isoform expression

To better understand genotype-specific differences in *Dmd* isoform expression, we compared their expression between WT and *mdx52* mice. Neither *Dmd* isoforms nor regional probes showed statistically significant differences at the sample level (*n* = 3) (Figure 2A). However, exon 30–44 regional probes, which included a larger number of probe sets per region than isoform probes, leading to a stronger signal, showed a trend of lower expression in *mdx52* mice compared to WT mice (Figure S13A). We therefore examined the expression of the exon 30–44 regional probe, which was highly associated with the spatial expression pattern of the full-length isoforms, across brain regions and cell types (Figures S13B–S13D).

Across brain regions, we observed that the expression of the full-length isoforms tended to decrease in the *mdx52* group. In the isocortex, the full-length isoforms had significantly lower expression in cortical layers 2/3, 4, 5, and 6b. In the hippocampal formation, the full-length isoforms showed a downward trend in the CA2/3 region of *mdx52* mice. For other brain regions, the full-length isoforms showed significantly lower expression in the olfactory areas, basolateral, central, and medial amygdalar nucleus, caudo-putamen, and ventricular system in the *mdx52* group (Figure 2D).

Regarding cell types, we observed that the full-length isoforms differed significantly in neuronal cell types in the *mdx52* brain. This included IT-ET Glut, NP-CT-L6b, CTX-CGE GABA, CTX-MGE GABA, CNU-LGE GABA cells, which are primarily located in the isocortex, hippocampus, and basolateral amygdalar nucleus. Among non-neuronal cell types, we observed a significant difference in OPC-Oligo cells (Figure 2E). Our data also indicate reduced expression of full-length isoforms among the abovementioned cell types in the *mdx52* group (Figure 2F). Interestingly, the observed changes in the expression of the full-length isoforms were not explained by differences in cell type proportions between WT and *mdx52* mice (Figure 2G).

### Spatial divergence and coexpression of full-length and shorter *Dmd* isoforms in the wildtype mouse brain

We next sought to identify regions and cell types showing mutually exclusive expression or coexpression of full-length and shorter *Dmd* isoforms in the WT mouse brain. For this purpose, we focused on promoter-specific probes, as only these could detect the expression of specific isoforms. We categorized *Dmd* isoforms into two groups: the full-length isoforms (Dp427c/m/p1), excluding isoform Dp427p2 due to its low expression, and shorter isoforms (Dp71 + Dp40, Dp40).

**Figure 3:**
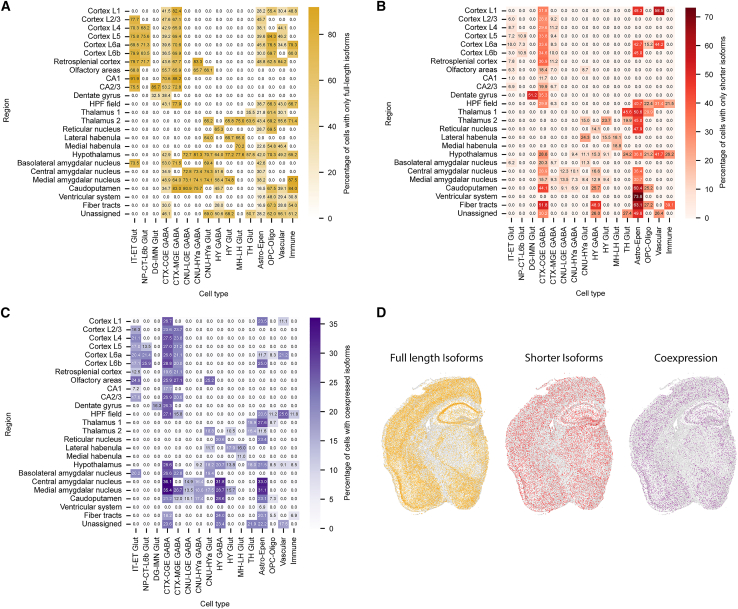
Localization and coexpression of the full-length and shorter *Dmd* isoforms in the wildtype mouse brain (A–C) Heatmap shows the percentage of cells with (A) exclusive expression of full-length *Dmd* isoforms (Dp427c/m/p1), (B) exclusive expression of shorter *Dmd* isoforms (Dp71+Dp40, Dp40), and (C) coexpressed isoforms across regions and cell types in the wildtype mouse brain. (D) Spatial distribution pattern of cells exclusively expressing full-length or shorter *Dmd* isoforms, and cells coexpressing these *Dmd* isoforms in wildtype #1 mouse brain.

Cells expressing only the full-length *Dmd* isoforms were predominantly found among neuronal populations, comprising 65%–90% of all *Dmd*-positive cells. These included IT-ET Glut, NP-CT-L6b Glut, and CTX-MGE GABA cells, which are primarily located in the isocortex, hippocampal CA region, and amygdala. Cells expressing only the full-length *Dmd* isoforms included CNU-LGE GABA, CNU-HYa GABA, and CNU-HYa Glut populations, distributed across the thalamus, hypothalamus, amygdala, and caudoputamen. Notably, cells expressing only the full-length *Dmd* isoforms were also abundant among OPC-Oligo populations (Figures 3A, 3D, S14A, and S14B).

Cells with exclusive expression of the shorter *Dmd* isoforms were found among both neuronal and non-neuronal populations. We observed approximately 50% of *Dmd* isoform-positive DG-IMN Glut cells in the dentate gyrus and around 30% of CTX-CGE GABA *Dmd* isoform-positive cells across various brain regions expressed only the shorter isoforms. Additionally, our data revealed that Astro-Epen cells also exhibited exclusive expression of shorter isoforms, comprising 35%–74% of *Dmd* isoform-positive cells within this population (Figures 3B and 3D).

Next, we investigated the proportion of cells coexpressing both the full-length and shorter *Dmd* isoforms. These cells were predominantly located in the isocortex, hippocampus, and amygdala. Notably, CTX-CGE GABA neurons showed a relatively high prevalence of coexpression, representing approximately 25% of the *Dmd*-positive population within this subtype. A similar pattern was also observed in IT-ET Glut and CTX-MGE GABA cells, but with lower coexpression rates (Figures 3C and 3D). This suggests that the coexpression between full-length and shorter *Dmd* isoforms is mainly driven by certain cell types (Figures 3C and 3D).

### Efficacy of exon 51-skipping events in the brain after therapeutic intervention

**Figure 4:**
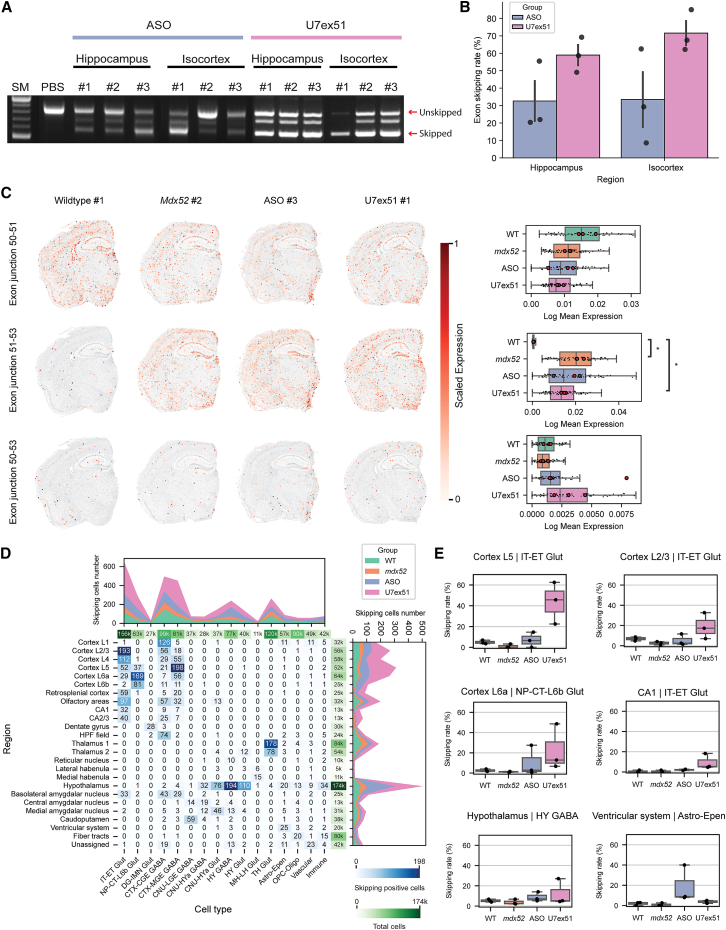
Exon-skipping events in the brain induced by exon 51-skipping treatments (A) RT-PCR gel illustrates exon 51 skipping in the hippocampus and isocortex of treated *mdx52* mice (all treated mice for ASO and U7ex51, *n* = 3, with 1 PBS-treated mouse as a representative control). SM: Size Marker. (B) Quantification of exon 51 skipping by TaqMan RT-qPCR in hippocampus and isocortex across ASO and U7ex51 samples. Results are shown as mean ± SEM. Statistical analysis was performed using Welch’s *t* test with BH correction at the sample-level comparison. (C) (Left) Spatial distribution of cells expressing exon-exon junction probes representing unskipped transcripts, exon 52 deletion, and combined exon 52 deletion with exon 51 skipping. (Right) Boxplot with overlaid dot plots showing the log-transformed mean expression of each exon-exon junction probe across samples and brain regions (black) and the log-transformed mean expression per mouse in WT and *mdx52* groups (red). Dots are displayed only within the interquartile range (Q1–Q3) of each boxplot. Statistical analysis was performed using Welch’s *t* test with BH correction at the sample level. ∗*p* < 0.05. (D) Frequencies of exon 51 skipping events across brain regions and cell types for all samples in each group. (E) Boxplot shows exon 51 skipping rates in Xenium data per sample in selected brain regions and cell types. Statistical analysis was performed using Welch’s *t* test with BH correction at the sample-level comparison.

To evaluate exon 51-skipping therapies in the DMD brain, *mdx52* mice were treated with two distinct approaches (ASO and U7ex51-mediated exon skipping). Exon 51 skipping was verified using RT-PCR in the isocortex and hippocampus (Figure 4A). To better align the results from RT-PCR quantification, we calculated the exon-skipping rate as the proportion of skipped transcripts out of the total transcripts (skipped plus unskipped) in the tissue. We observed that samples under U7ex51 treatment induced a higher level of exon 51 skipping in both regions relative to ASO treatment, although the differences were not statistically significant. This difference is likely attributable to both the distinct therapeutic strategies (U7ex51 versus ASO) and the timing of administration, since U7ex51 was delivered at the neonatal stage whereas ASO treatment was performed in adult mice (Figure 4B).

To spatially resolve exon 51-skipping events in the brain, we designed exon-exon junction probes targeting the *Dmd* transcript for Xenium: exon 50–51 junction (unskipped transcripts), exon 51–53 junction (exon 52 deleted transcripts), and exon 50–53 junction (skipped transcripts lacking both exons 51 and 52) (Figure 4C). We observed a trend of higher expression of the exon 50–51 junction in the WT group than in the *mdx52* groups, consistent with the reduced expression of *Dmd* transcript in *mdx52* mice. Exon 51–53 showed significantly higher expression in the *mdx52* group, with or without U7ex51 injection, compared to the WT group. This observation confirmed the specificity of the probe detecting the deletion of exon 52. For the exon 50–53 junction probes, we observed a trend of higher expression in both ASO and U7ex51 compared to the *mdx52* group. However, sample-level comparisons showed that this difference was not statistically significant. Overall, these observations are similar to the RT-PCR results, supporting the robustness and specificity of the exon junction probes (Figures 4C, S15, and S16).

We next mapped exon 51-skipping events across brain regions and cell types and observed that they occurred predominantly in the neuronal population (Figure 4D). Cortical neurons exhibited abundant skipping events, as well as TH Glut cells in the thalamus, and both HY GABA and HY Glut cells in the hypothalamus. We also observed exon 51 skipping in neuronal cells within the hippocampal CA regions and the amygdala, but these events were less frequent, despite the high expression levels of full-length *Dmd* isoforms typically observed in these brain regions in WT mice (Figure 2B).

Furthermore, we examined specific brain regions and cell types to compare skipping rates between therapeutic groups (Figure 4E). Unlike RT-PCR, which quantifies bulk transcript ratios, we defined the skipping rate in our Xenium data as the proportion of cells expressing the skipped junction (Exon 50-53 junction) relative to the total number of cells expressing any targeted dystrophin junction (see Methods). Our data showed that both treated brains had an increased trend of skipping rates relative to the untreated *mdx52* brains in cortical neurons, such as IT-ET Glut in cortex L2/3 and L5, NP-CT-L6b Glut in cortex L6, and HY GABA in the hippocampus. Overall, widely elevated skipping rates were identified in the U7ex51 group with P1 administration. In addition, we also found that the ASO group had a higher skipping rate in the astrocyte-ependymal cluster, driven primarily by ependymal cells within the ventricular system, suggesting that the occurrence of exon 51-skipping events may be cell type specific (Figure S17). Surprisingly, despite high Dp427c expression in the IT-ET Glut of the CA1 region, both therapeutic groups exhibited an overall lower skipping rate. This observation indicates that the spatial distribution of exon 51 skipping does not strictly correlate with the regions or cell types enriched for the full-length *Dmd* isoforms.

### Dp427-sized isoform restoration in exon 51-skipped cells

**Figure 5:**
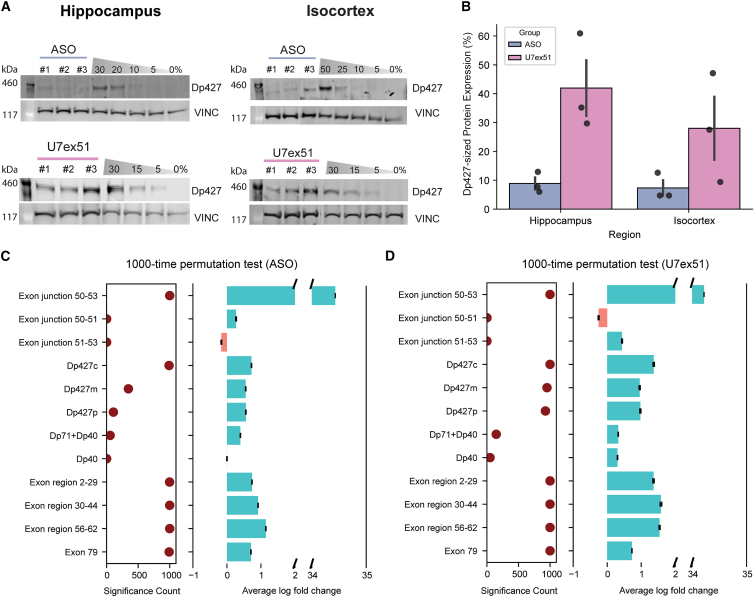
Restoration of Dp427-sized isoform at protein and transcript level after exon 51-skipping therapy (A) Quantification of Dp427-sized dystrophin restoration levels by western blot in ASO- and U7ex51-treated samples. Protein levels were quantified using standard curves generated on separate gels. Each gel included 0%–50% wildtype lysate dilutions mixed with *mdx52* lysate. VINC: vinculin. (B) Percentage of Dp427-sized isoform dystrophin restoration in the hippocampus and cortex across ASO and U7ex51 treatment groups, measured by western blot. Results are shown as mean ± SEM. Statistical analysis was performed using Welch’s *t* test with BH correction. (C and D) Differential expression analysis of *Dmd* transcripts between exon 51-skipped and matched unskipped cells in (C) ASO-treated and (D) U7ex51-treated groups, assessed using the Wilcoxon test with 1,000 permutations. The dot plot displays the number of significant outcomes across 1,000 permutations, while the bar plot shows the average log fold change ±SEM in exon 51-skipped cells across permutations.

Given the detectable induction of exon skipping by both therapeutics in our study, we next assessed whether the exon-skipping events led to the restoration of Dp427-sized dystrophin in the hippocampus and isocortex by western blot (Figure 5A). We confirmed consistent restoration of the Dp427-sized isoform in both regions across all treated mice, regardless of the therapeutic approach. The ASO group showed an average of 8.9% ± 2.1% Dp427-sized dystophin restoration relative to WT levels in the hippocampus and 7.3% ± 4.6% in the isocortex (mean ± SEM). In comparison, the U7ex51 group showed a higher Dp427-sized dystrophin restoration with an average of 41.9% ± 9.6% and 28.0% ± 10.9% in the hippocampus and isocortex relative to the WT level, respectively (mean ± SEM).

To determine whether changes in Dp427-sized isoform expression were associated with exon 51-skipping events, we compared the expression of all *Dmd*-related transcripts between exon 51-skipped and non-skipped cells within each therapeutic group using a permutation-based matching strategy, which showed robust results in a sensitivity analysis (Figure S18). Among the 811 cells showing exon 51-skipping events in the ASO group (representing 0.4% of all cells in this group), we observed robustly elevated the expression of the Dp427c isoform and all *Dmd* regional transcripts among the exon 51-skipped cells, indicating restoration of the Dp427-sized isoform at the transcript level following exon 51 skipping (Figure 5C). In the U7ex51 group, we observed 1299 cells presenting exon 51-skipping events (representing 0.6% of all cells in this group). All Dp427-sized isoforms (Dp427c, Dp427m, and Dp427p), along with all regional *Dmd* transcripts, showed robust upregulation among the exon 51-skipped cells, indicating widespread Dp427-sized isoform restoration at the transcript level after U7ex51 administration at P1 (Figure 5D).

### ASO treatment induces regionally localized microglial activation

**Figure 6:**
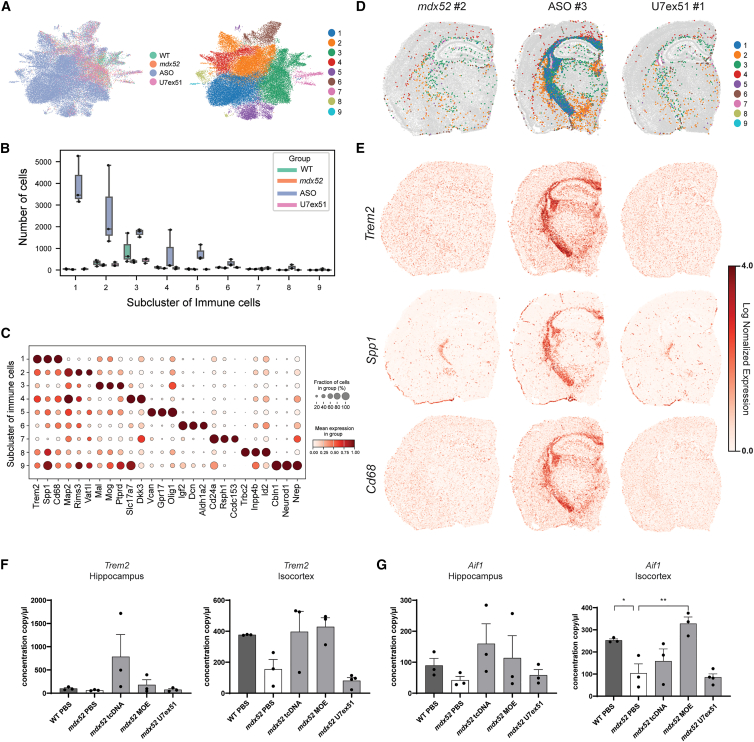
Immune response in ASO-treated brains of *mdx52* mice (A) UMAP visualization of subclusters of immune cells across all samples. (B) Boxplot with overlaid dot plot shows cell counts for each immune cell subcluster across all samples. (C) Dot plot shows marker gene expression profiles for each immune cell subcluster. (D) Spatial distribution of immune cell subclusters in tissue samples from *mdx52*, ASO-treated, and U7ex51-treated mice. (E) Spatial expression patterns of *Spp1*, *Cd68*, and *Trem2* in samples from *mdx52*, ASO-treated, and U7ex51-treated mice (log-normalized). (F and G) Droplet digital PCR (ddPCR) quantification of RNA levels for (F) *Trem2* and (G) *Aif1* across the hippocampus and isocortex in samples from WT, *mdx52*, ASO-treated (tcDNA and 2′-*O*-methoxyethylribose [MOE]), and U7ex51-treated mice. Results are expressed as copies per μL of input RNA and shown as mean ± SEM. Data normality was assessed prior to statistical testing; one-way ANOVA test was applied when assumptions were met, otherwise a non-parametric Kruskal-Wallis test was used. ∗*p* < 0.05 and ∗∗*p* < 0.01.

Previous study have indicated that ICV-administered ASOs can trigger persistent, chemistry-dependent immune responses in the rodent brain.^40^ We further examined immune cell populations to assess whether exon 51 skipping could induce an immune response in the CNS. We first identified immune cell subpopulations by re-clustering all annotated immune cells with unsupervised clustering, resulting in 9 immune subpopulations (Figure 6A). We noticed an overall expansion of immune cells in ASO-treated samples (Figure 6B). Next, we focused on a specific subcluster (Cluster 1), characterized by the expression of *Trem2*, *Spp1*, and *Cd68* (Figure 6C), which was predominantly enriched in the ASO-treated group (Figure 6B). The marker genes of this subcluster suggest an increase in immune cells exhibiting an activated microglial phenotype.^41^^,^^42^^,^^43^

We further projected all subclustered immune cells spatially in samples from *mdx52*, ASO-treated, and U7ex51-treated groups (Figure 6D). Cluster 1, identified as activated microglial cells based on its gene markers, was specifically localized to the fiber tract and hypothalamic regions in ASO-treated samples, consistent with the spatial expression patterns of *Trem2, Spp1*, and *Cd68* (Figure 6E). To determine whether this immune response is specific to the ASO chemistry used in this study (tcDNA), we further analyzed the expression of immune markers in additional *mdx52* samples treated with ASOs modified with the 2′-*O*-methoxyethylribose (MOE) chemistry using droplet digital PCR (ddPCR) (Figures 6F, 6G, and S19). We observed similarly elevated expression of immune markers *Trem2* and *Aif1,* and also increased mRNA levels of Tnf-α and *Cxcl10* in the MOE-treated samples, suggesting that the distinct immune dynamics may be associated with ASO treatment in general, rather than being specific to a particular chemical backbone.

## Discussion

This study provided a detailed spatial map of *Dmd* isoform expression in the mouse CNS, alongside an evaluation of exon-skipping therapies, including ASO administration at P49 and U7ex51 administration at P1. Our findings revealed distinct expression and coexpression patterns among full-length and shorter *Dmd* isoforms. We also revealed spatial patterns of induced responses to exon 51 skipping, including restoration of Dp427-sized isoforms at the transcript and protein levels, with changes in immune cell dynamics after ASO administration at P49. Rather than making definitive claims regarding the efficacy of specific modalities, our objective was to highlight the utility of this high-resolution spatial technology in characterizing differences between therapeutic approaches, providing a foundation for future research.

Identifying multiple *Dmd* isoforms in the brain remains challenging. Although recent advances have introduced *in situ* hybridization and sequencing-based approaches, both methods face significant limitations: *In situ* hybridization is constrained by the multiplexing capacity, while sequencing techniques are restricted by the long *Dmd* transcript lengths and low abundance in the brain, making accurate isoform mapping difficult. In this study, we provide the first detailed spatial mapping of multiple *Dmd* isoforms in the mouse brain across annotated brain regions and cell types at single-cell resolution using Xenium with custom probes.

We observed two distinct spatial expression patterns between the full-length and shorter *Dmd* isoforms . In the hippocampus, full-length isoforms were primarily expressed in IT-ET Glut cells of the CA region, while shorter isoforms were enriched in the granule neurons in the dentate gyrus, consistent with previous findings.^44^ Interestingly, we observed distinct expression patterns across cortical layers: Full-length *Dmd* isoforms were expressed at higher levels in pyramidal neurons located in deeper cortical layers, while shorter isoforms were enriched in GABAergic interneurons and astrocytes in layer 1, suggesting that *Dmd* isoform expression is specific to both cell type and cortical layer. Previous studies have demonstrated that full-length *Dmd* isoforms play an important role in regulating the organization of gamma-aminobutyric acid type A receptors (GABA_A_Rs) and inhibitory synaptic transmission in the CNS.^45^ In the sensorimotor cortex of *mdx* mice, loss of Dp427 leads to a redistribution of GABA_A_R subunits, with increased α1 and decreased α2 expression as well as a higher proportion of small α5-containing clusters.^46^ α5-containing GABA_A_Rs are indicated to mediate tonic inhibition in layer 5 pyramidal neurons of the rat cortex.^47^ These results suggest that loss of Dp427 might disrupt the tonic inhibition of deep cortical layer pyramidal neurons, which may contribute to the motor and cognitive abnormalities observed in dystrophinopathies. Further investigation into the association between full-length isoforms and GABA_A_R subunits in deeper cortical layers might provide more critical insights into the molecular mechanisms in DMD.

Despite the overall distinct expression patterns of full-length and shorter *Dmd* isoforms, we observed multiple neuronal cell types coexpressing these isoforms, particularly that one-third of *Dmd*-positive CTX-CGE GABA neurons showed the coexpression of long and short isoforms. The coexpression of isoforms in these neurons might suggest a potential interplay or regulation. Previous studies have reported rare instances of *Dmd* isoform coexpression in neurons.^14^ Our approach of capturing cells across broad brain regions indicates more frequent coexpression events in specific neuronal cell types. These findings suggest the notion of a cell-specific transcriptional program rather than local proximity or cellular neighborhood.

Our targeted spatial transcriptomics approach allowed the evaluation of transcript responses in two different exon-skipping therapies targeting *Dmd* exon 51. Both approaches successfully induced exon 51-skipping transcripts in the brain. U7ex51 administration at postnatal day 1 showed high skipping efficiency and robust transcript signal restoration across all Dp427-sized isoforms, whereas ASO administration at postnatal day 49 reliably restored only the Dp427c-sized isoform at the transcript signal under our permutation test. The broad Dp427-sized isoform restoration upon neonatal U7ex51 administration is likely attributable to the earlier intervention window as well as the persistence and accumulation of antisense sequences produced from the AAV genome. In this study, our data are limited by the small number of samples and differences in to the timing of intervention. Additionally, our current conclusions are restricted to Dp427-sized isoform restoration at transcript and protein levels. Despite these limitations, our data provide encouraging evidence on the spatial distribution of the molecular response in both neonatal administration of U7ex51 and adult ASO administration. Future work with adequately powered cohorts will be particularly valuable to determine whether the enhanced molecular and protein restoration observed after neonatal U7ex51 administration translates into improved behavioral outcomes, potentially exceeding the partial behavioral rescue previously achieved with adult ASO administration.^38^^,^^48^

Interestingly, although the CA1 region is enriched in full-length *Dmd* isoforms, it exhibited relatively low levels of exon 51 skipping. This contrasts with previous findings, which reported approximately 30% exon skipping in CA1 after seven weeks of treatment with tcDNA-ASO,^38^ possibly due to inherent variability in treatment distribution or the limited sample size (*n* = 3) in this study.

In our ASO-treated groups, we observed the activation of a distinct microglial population in the brain. Follow-up analyses in 2′MOE-injected animals indicated that this response was not specific to a given chemical modification (tcDNA versus MOE), suggesting that microglial activation may represent a general response to ASO administration rather than to a particular ASO backbone. This observation is consistent with previous reports of microglial activation following CNS administration of 2′-*O*-methyl ASOs^49^ and other ASO therapies,^50^ which collectively point to an immune reaction elicited by ASO-based therapies in the brain. Such effects should be carefully considered in the design and development of future CNS-targeted treatments. In contrast, U7ex51-treated samples did not display obvious signs of microglial activation, suggesting a potential advantage of this vectorized approach. However, we cannot exclude that the earlier timing of U7ex51 administration also contributed to this difference. During early postnatal development, microglia occupy a distinct transcriptional state enriched for neurodevelopmental and tissue-remodeling functions, which precedes full maturation of immune sensing programs.^51^^,^^52^ Therefore, part of the differential microglial activation may reflect timing of intervention rather than treatment-specific effects.

In summary, we provide a detailed map of cell type and region specific expression of *Dmd* isoforms in the mouse brain and investigate how these patterns differ in the *mdx52* mice, which lack the full-length and Dp140 isoforms. Additionally, we evaluated the spatial distribution of molecular response of two different exon 51-skipping therapies in the CNS after Dp427-sized isoform(s) restoration. Our findings demonstrate the power of high-resolution spatial transcriptomics to resolve isoform-level complexity *in situ*, supporting its broader application in studying *Dmd* transcript diversity in the brain and guiding the development of future therapeutic strategies.

### Limitations of the study

Several limitations should be acknowledged regarding our analysis. First, the small sample size limits comparisons at the individual level, preventing robust conclusions from group comparisons of *Dmd* isoform reduction, exon 51 junction expression, and skipping rates across regions and cell types.

Second, the comparison between the two therapeutic strategies is confounded by differences of timing of administration and likely treatment persistence (P49 for ASO versus P1 for U7ex51). The observed differences therefore cannot be attributed solely to therapeutic modality, but these could also be associated with timing of administration and the prolonged persistence of AAV-mediated expression.

Third, although previous work that partial restoration of Dp427-sized dystrophin in the mouse brain following exon skipping treatment was accompanied by behavioral improvement, the quantitative relationship between region or cell type-specific molecular restoration of the dystrophin isoform and functional gain remains unclear.^38^^,^^48^ Building on the molecular restoration and its spatial distribution revealed here, further behavioral studies are needed to address this question.

Fourth, we were unable to include Dp140 in downstream analyses because of its low detection signal. A similar situation was observed for exon junction probes, for which relatively lower exon-skipping signals remained after filtering compared to the transcript levels in RT-PCR. This is a technological limitation likely resulting from the reduced brightness of single probe sets, which are nevertheless required to distinguish the Dp140 5′ UTR due to its limited sequence space (Table S1). Future studies that bridge this technological gap and describe the spatial and cellular expression of Dp140 will help clarify previous literature reporting how this isoform is associated with impairments in verbal memory, attention, and executive function.^13^^,^^53^ Recent studies have also shown that loss of Dp140 is linked to ASD-like behaviors and enhanced anxiety in the *mdx52* mice.^54^^,^^55^

In addition, the cerebellum was not included because the limited Xenium slide size restricted the analysis to a single coronal section encompassing the cortex, hippocampus, amygdala, and thalamus. Owing to its anatomical distance from these regions, the cerebellum could not be captured, despite its association with verbal working memory and motor deficits in DMD,^56^^,^^57^ and its high dystrophin expression, including Dp427p located in the Purkinje cells.^14^^,^^58^ Future comparisons of classic *mdx* mice, where Dp427 is disrupted while shorter isoforms such as Dp140 are preserved, would help disentangle Dp427-driven effects from Dp140-associated contributions to CNS.

Regarding the observed elevated immune cell population in the ASO-treated group, we acknowledge that these interpretations of immune spatial patterns remain exploratory, constrained by the small sample size (*n* = 3) and a restricted gene panel. The targeted panel in particular may limit detailed annotation of immune subpopulations.

Finally, the Xenium panel used in our work allows the detection of only a limited number of genes, restricting deeper cell type annotation. Future analyses with larger gene panels will enable more comprehensive investigations, and spatial proteomics on matched tissue samples will be needed to evaluate how *Dmd* isoform gene expression relates to protein isoform expression.

## Resource availability

### Lead contact

Requests for further information and resources should be directed to and will be fulfilled by the lead contact: Pietro Spitali (p.spitali@lumc.nl).

### Materials availability

This study did not generate new unique reagents.

### Data and code availability

• Raw and processed Xenium data have been deposited in NCBI’s Gene Expression Omnibus and are accessible through Series accession number GSE318507 (https://www.ncbi.nlm.nih.gov/geo/query/acc.cgi?acc=GSE318507) and are publicly available as of the date of publication.
• As a reference for cell type annotation, adult mouse scRNA-seq (v2 chemistry) from the Allen Brain Atlas can be downloaded from the public data repository (https://allen-brain-cell-atlas.s3.us-west-2.amazonaws.com/index.html#expression_matrices/WMB-10Xv2/20230630/).
• All analysis scripts and code used for data preprocessing and visualization are provided in a public GitHub repository (https://github.com/Qirongmao97/Xenium_DMD_brain) and are publicly available as of the date of publication.
• Any additional information required to reanalyze the data reported in this paper is available from the corresponding authors upon request.

## Acknowledgments

We acknowledge funding by the Medical Genomics Theme and the Human Genetics Department of the LUMC. We thank Dr. Katsuki Motoya, Prof. Sasaoka Toshikuni (Department of Comparative and Experimental Medicine/Brain Research Institute; Niigata University, Japan), Dr. Jun Tanihata and Dr. Shin’ichi Takeda (National Center of Neurology and Psychiatry, Tokyo, Japan) for providing the mdx52 mouse breeders. We also thank Sandra de Haan (Leiden University Medical Center; Leiden, The Netherlands) for reviewing the manuscript and Lola van Doorne for helping perform the Xenium experiment. Samples from *mdx52* mice were collected as part of a project funded by the European Union’s Horizon 2020 research and innovation program “Brain Involvement iN Dystrophinopathies” under grant agreement no. 847826. It was also supported by Institut National de la Santé et de la Recherche Médicale (Inserm), Université Paris-Saclay (France) and Paris Ile-de-France Region.

## Author contributions

Q.M. conceptualized the study, performed most of the data analysis, and prepared the original manuscript draft. A.A. contributed to Xenium data annotation analysis. O.V. and M.D. contributed to qPCR/ddPCR experiments and subsequent data analysis. M.D. also performed RNA extraction. S.D.V. and L.H. prepared samples for the Xenium experiment. A.G. provided mouse brain samples and assisted with methodology and funding acquisition. P.S. and A.M. managed the project and contributed to methodology, while P.S., A.M., M.V.P., and A.A.R. jointly supervised the work, led by P.S. and A.M. All authors participated in reviewing and editing the manuscript and approved the final version.

## Declaration of interests

AAR discloses being employed by LUMC, which has patents on exon skipping technology, some of which have been licensed to BioMarin and subsequently sublicensed to Sarepta. As co-inventor of some of these patents, AAR was entitled to a share of royalties. AAR further discloses being an ad hoc consultant for PTC Therapeutics, Sarepta Therapeutics, Regenxbio, Dyne Therapeutics, Lilly, BioMarin Pharmaceuticals Inc., Eisai, Entrada, Takeda, Splicesense, Galapagos, Sapreme, Italfarmaco and AstraZeneca. AAR also reports being a member of the scientific advisory boards of Hybridize Therapeutics (past), Silence Therapeutics, Sarepta Therapeutics, Sapreme and Mitorx. Remuneration for consulting and advising activities is paid to LUMC. In the past 5 years, LUMC also received speaker honoraria from Alnylam Netherlands, Italfarmaco and Pfizer and funding for contract research from Sapreme, Eisai, BioMarin, Galapagos and Synaffix. Project funding is received from Sarepta Therapeutics and Entrada via unrestricted grants. All other authors declare no competing interests.

## STAR★Methods

### Key resources table

**Table:** 

REAGENT or RESOURCE	SOURCE	IDENTIFIER
**Antibodies**		

Anti-Dystrophin antibody [EPR9598 (ABC)] (rabbit monoclonal)	Abcam	Cat# ab154168; RRID:AB_2858227
Anti-Vinculin antibody, clone hVIN-1 (mouse monoclonal)	Sigma-Aldrich	Cat# V9131; RRID:AB_477629
IRDye® 800CW Goat anti-Mouse IgG (H + L) secondary antibody	LI-COR Biosciences	Cat# 926–32210; RRID:AB_621842
IRDye® 680RD Goat anti-Rabbit IgG (H + L) secondary antibody	LI-COR Biosciences	Cat# 926–68071; RRID:AB_10956166

**Bacterial and virus strains**		

Self-complementary AAV serotype 9 vector encoding modified U7snRNA designed to target dystrophin exon 51 (scAAV9-U7-51M)	Vacca et al.^48^	N/A

**Chemicals, peptides, and recombinant proteins**		

Tissue-Tek® O.C.T. Compound	Sakura Finetek	Cat# 4583
TRIzol™ Reagent	Thermo Fisher Scientific/Invitrogen	Cat# 15596026
RIPA Lysis and Extraction Buffer	Thermo Fisher Scientific	Cat# 89901
Custom synthetic gBlock standards corresponding to *Dmd* exons 49–54 with deletion of exon 52 (Ex49–54Δ52; mdx52 transcript) or exons 51–52 (Ex49–54Δ51 + 52; exon 51-skipped mdx52 transcript	Integrated DNA Technologies	N/A
NuPAGE™ 3–8% Tris-Acetate protein gels	Invitrogen/Thermo Fisher Scientific	Cat# 12035665

**Critical commercial assays**		

Xenium Mouse Brain Gene Expression Panel (2 rxns)	10x Genomics	Cat# 1000462
Xenium Custom Gene Expression Panel (up to 50 genes)	10x Genomics	Cat# 1000464
LunaScript® RT SuperMix Kit	New England Biolabs	Cat# M3010L, M3010X, M3010E
GoTaq® G2 Colorless Master Mix	Promega	Cat# M7833
ddPCR Supermix for Probes (No dUTP)	Bio-Rad	Cat# 186-3024
Pierce™ BCA Protein Assay Kit	Thermo Fisher Scientific	Cat# 23227

**Deposited data**		

Allen Brain Reference Atlas (The Mouse Whole Brain Atlas)	Allen Institute for Brain Science	https://alleninstitute.github.io/abc_atlas_access/descriptions/WMB_dataset.html
Xenium raw/processed data from this study	This paper	GEO: GSE318507

**Experimental models: Organisms/strains**		

*mdx52* mouse (*Dmd* exon 52 deletion; C57BL/6J background)	Araki et al.^59^	N/A

**Oligonucleotides**		

Tricyclo-DNA antisense oligonucleotide targeting mouse *Dmd* exon 51	Saoudi et al.^54^^,^^55^	N/A
Ex-m50F primer 5′-AGGAAGTTAGAAGATCTGAGG-3′	This paper	N/A
Ex-m55R primer 5′-GGAACTGCTGCAGTAATCTATGA-3′	This paper	N/A
TaqMan assay for exon 50–51 junction	Integrated DNA Technologies	Mm.PT.58.41685801
Custom TaqMan assay for exon 50–53 junction Forward 5′-GCACTACTGGAGCC TTTGAA-3′; Reverse 5′-TTCCAGCCATTGTGTTGAATC-3′; Probe 5′-ACAGCTGCAGAACAGGAGACAACA-3′	Integrated DNA Technologies	N/A
Summary of ddPCR Assays and Reagents	See Table S4	

**Software and algorithms**		

Xenium Ranger v1.7.1	10x Genomics	https://www.10xgenomics.com/support/software/xenium-ranger/latest
Xenium Explorer v3.0	10x Genomics	https://www.10xgenomics.com/support/software/xenium-explorer/latest
BANKSY v0.1.6	Singhal et al.^60^	https://github.com/prabhakarlab/Banksy_py
scvi-tools v1.1.4	Gayoso et al.^61^^,^^62^	https://github.com/scverse/scvi-tools
Scanpy v1.11.5	Wolf et al.^63^	https://scanpy.scverse.org/en/stable/
Squidpy v1.6.5	Palla et al.^64^	https://squidpy.readthedocs.io/en/stable/
scanpro v0.3.2	Alayoubi et al.^65^	https://github.com/loosolab/scanpro
R v4.3.2	R Core Team^66^	https://www.r-project.org/
Python v3.10	Python Software Foundation^67^	https://www.python.org/
GraphPad Prism v10.3.3	GraphPad Software Inc.	https://www.graphpad.com/
Image Studio Lite v4.0.21	LI-COR Biosciences	https://www.licorbio.com/image-studio
QuantaSoft Analysis Pro, QX Manager Software 2.2 standard edition	Bio-Rad Laboratories	RRID:SCR_025321
Xenium analysis code	This paper	https://github.com/Qirongmao97/Xenium_DMD_brain

### Experimental model and study participant details

#### Animals, injections and tissue collection

The *mdx52* mouse model, carrying a deletion of exon 52 in the *Dmd* gene, was developed by Dr. Katsuki Motoya’s team.^59^ This model was created by replacing exon 52 with a neomycin resistance gene, resulting in the absence of Dp427, Dp260, and Dp140 dystrophins, while maintaining the expression of Dp116, Dp71, and Dp40. The strain was backcrossed with C57BL/6J mice for over eight generations to ensure genetic stability. This mouse line was generously shared by Prof. Toshikuni Sasaoka (Department of Comparative & Experimental Medicine/Brain Research Institute, Niigata University, Japan). Breeding pairs were provided to our facility based at UVSQ in Montigny-le-Bretonneux (France), by Dr. Jun Tanihata and Dr. Shin’ichi Takeda (National Center of Neurology and Psychiatry, Tokyo, Japan). Heterozygous females were bred with *mdx52* males to produce *mdx52* males and their C57BL/6J wildtype (WT) littermates. Genotyping was performed using PCR analysis of tail DNA. All animal care and experimental protocols adhered to European regulations (CEE 86/609/EEC and EU Directive 2010/63/EU) as well as French national guidelines (87/848) and were approved by the Paris Center- South Ethics Committee (no. 59).

Adult *mdx52* mice (P49, *n* = 3) were deeply anesthetized with a single intraperitoneal injection of ketamine (95 mg/kg) and medetomidine (1 mg/kg) and received bilateral intracerebroventricular (ICV) injections of tricyclo-DNA (tcDNA) antisense oligonucleotide (ASO) targeting *Dmd* exon 51 (400 μg total; 5 μL per hemisphere, infused at 0.5 μL/min).^38^ Age-matched *mdx52* mice (*n* = 3) and WT littermates (*n* = 3) received PBS under identical conditions as controls. To investigate whether earlier intervention might yield higher dystrophin rescue, a separate experimental group of three neonatal *mdx52* mice (P1, *n* = 3) were anesthetized by hypothermia and received bilateral ICV injections of a self-complementary AAV9 vector encoding a modified U7snRNA targeting exon 51 (scAAV9-U7-51M; 2 μL per hemisphere, 7 × 10^11^ vg total).^48^ Stereotaxic coordinates for adult ICV injections were −0.5 mm from bregma, 1 mm lateral, and −2 mm from dura, whereas for neonatal ICV injections, coordinates were positioned at an equal distance between lambda and bregma, 0.8–1 mm lateral to the sagittal suture, and 3 mm deep.^38^^,^^68^ Littermates were housed in groups of two to five per cage under a 12-h light/dark cycle (lights on at 7:00 a.m.) with *ad libitum* access to food and water. All mice were euthanized by cervical dislocation at 14 weeks of age. Brains were collected and processed as follows: one hemisphere was immediately frozen on dry ice for Xenium analysis, while the other hemisphere was dissected to isolate specific brain regions, including the hippocampus (HIP) and cortex (CX). These tissue samples were snap-frozen in liquid nitrogen and stored at −80 °C until further processing for RT-qPCR, ddPCR, and western blot experiments. All animals included in this study were male. Therefore, the influence of sex on experimental outcomes was not assessed.

### Method details

#### Xenium sample preparation

Fresh-frozen hemi-brain samples were equilibrated to cryostat temperatures (−20 °C) for 30 min before mounting with Tissue-Tek O.C.T. Compound (Sakura Finetek, Torrance, CA, USA) using fast freezing. Samples were cryo-sectioned at 10 μm using Leica CM3050 S cryostat (Leica Microsystems, Wetzlar, Germany) in coronal orientation and placed onto Xenium slides with randomization of placement, followed by fixation and permeabilization. Experiments were performed according to the manufacturer’s instructions (10x Genomics, CG000579 & CG000581). A total of 247 genes from the Xenium pre-designed brain panel and 50 genes from a custom panel were then applied to the slides (Table S1). All probes hybridized to their target RNAs and were ligated to form circular DNA, and enzymatically amplified to generate multiple copies of gene-specific barcodes for each transcript. Background autofluorescence was quenched, and nuclei were counterstained with DAPI, following the manufacturer’s protocol (10x Genomics, CG000582). Processed Xenium slides were loaded into the Xenium analyzer (v1.8.2.1).

#### Xenium data analysis

Raw data were processed using Xenium Ranger (v1.7.1). Cell segmentation was based on DAPI-stained nuclei with a 15 μm expansion. Low-quality transcript signals (q < 20) were excluded from downstream analysis. Cells were assigned manually to corresponding samples using Xenium Explorer (v3.0).

Xenium data were then annotated at two levels: brain region and cell type. For brain region annotation, we used BANKSY v0.1.6^60^ with lambda = 0.8 to group the cells on the first Xenium slide into different brain regions. These regions were then visually matched to the anatomy map from the Allen Brain Atlas^69^ based on the closest coronal section. Then, we used scANVI with scvi-tools v1.1.4^61^^,^^62^ to transfer the labels from the first slide to the other three slides that were not yet annotated. For cell type annotation, we used single-cell transcriptomics data (v2 chemistry) from the whole adult mouse brain from the Allen Brain Cell Atlas as a ref.^39^ First, we selected data from brain regions that matched those in our Xenium data, as identified in the region annotation described earlier. Then, we removed cell types with low expression levels and downsampled the remaining data to 10% for each cell type to reduce computational load. Finally, we transferred the cell type labels from the reference dataset to our Xenium data using scANVI (Figure S11; Tables S2 and S3).

#### Differential proportion analysis

We applied Scanpro v0.3.2 to compare cell type proportions between groups.^65^ First, we calculated the proportion of each cell type for every replicate within each group. Then, Scanpro was used to fit a linear model on these proportions across groups, using empirical Bayes shrinkage to stabilize the variance estimates. To account for the heteroskedasticity that can occur with proportions from a binomial distribution, Scanpro applied a logit transformation to all percentage values before testing, then using an empirical Bayes moderated *t* test when comparing two groups. Finally, we adjusted the resulting *p*-values for multiple testing using the Benjamini–Hochberg (BH) method.

#### Isoform localization and co-expression analysis

To calculate the (co)expression rates of the *Dmd* full-length and shorter isoforms across brain region and cell type pairs several steps were undertaken. First, to remove brain regions with very few or no cells of that type, we kept only regions where each cell type made up more than 0.5% of the cells based on the cell type proportion map across regions (Figure S4A). Second, we removed any region–cell type pairs where the isoform had fewer than 10 raw counts. Finally, for each region and cell type, we calculated the expression rate as the proportion of cells positive for each isoform, and the coexpression rate as the number of cells positive for both isoforms divided by the total number of cells positive for either isoform (i.e., the Jaccard index between cells expressing either one or both isoforms).

#### Skipping rate calculation in Xenium data

For each region and cell type in every sample, we calculated the exon 51 skipping rate as:

$$Exon\;51\;Skipping\;Rate\;\left(\%\right)=\frac{N_{exon\;50-53}}{\left(N_{exon\;50-51}+N_{exon\;51-53}+N_{exon\;50-53}\right)-N_{coexpressed}}\times 100\%$$

Here, $N_{exon\;50-51}$ is the number of cells expressing the unskipped probe, $N_{exon\;51-53}$ is the number of cells expressing the exon 52 deletion probe, $N_{exon\;50-53}$ is the number of cells expressing the exon 51-skipping probe. We subtracted $N_{coexpressed}$, the number of cells expressing multiple probes, to avoid double counting. If no cells were detected in these categories, the skipping rate was set to zero.

#### Differential expression analysis between exon 51-positive and exon 51-negative cells

To address the large imbalance in cell numbers between the exon 51-skipped positive and negative populations during differential expression analysis, we designed a permutation test. From the exon 51-skipped negative population, we selected a subset of cells that matched the positive population in terms of region and cell type distribution. If the pool of matching cells was not sufficient to match the size of the exon 51-skipped positive population, we expanded the selection pool further based on matching the cell type distribution alone. If that pool was also exhausted, we further relaxed the criteria to match only by region. In the ASO group, each cell in the exon 51-skipped negative population could be selected up to five times, whereas in the U7ex51 group, each cell could be selected up to seven times, due to the higher numbers of exon 51-positive cells and the need to maintain balanced sampling between positive and negative populations. We applied sc.tl.rank_genes_groups() from Scanpy v1.11.5 using the Wilcoxon test, with Benjamini-Hochberg correction for multiple testing.^63^ The permutation test was run 1,000 times. For each gene, we recorded the number of times it showed a significant difference and calculated the average log-fold change across all iterations as the effect size.

#### Western blot

Protein extracts were prepared from brain tissues using RIPA lysis and extraction buffers (Thermo Fisher Scientific, Waltham, MA, USA), supplemented with SDS to a final concentration of 5% (Bio-Rad, France). Protein concentrations were measured with the BCA Protein Assay Kit (Thermo Fisher Scientific, Waltham, MA, USA). Samples were denatured at 100 °C for 3 min, and 25 μg of protein per sample was loaded onto NuPAGE 3–8% Tris-acetate gels (Invitrogen), according to the manufacturer’s protocol. Dystrophin expression was analyzed using the anti-dystrophin monoclonal rabbit antibody (ab154168, Abcam, France), while vinculin served as a loading control and was detected with the hVin-1 monoclonal mouse antibody (V9131, Sigma, Saint-Louis, USA). Detection was performed with an IRDye 800CW goat anti-mouse secondary antibody and an IRDye 680RD goat anti-rabbit secondary antibody (Li-Cor, Germany), and bands were visualized using the Odyssey CLx imaging system (Li-Cor). Quantification was carried out with Image Studio software (Li-Cor), normalizing dystrophin signals to vinculin levels. Standard curves were generated for each brain region by mixing lysates from wildtype (WT) and *mdx52* control samples to obtain defined dystrophin percentages (0%, 2.5%, 5%, 10%, and 20% relative to WT tissues).

#### RNA analyses

Total RNA was isolated from dissected brain structures using TRIzol reagent according to the manufacturer’s instructions (Thermo Fisher Scientific). For visualization of exon-skipping efficacy on gels, 1 μg of total RNA was subjected to RT-PCR analysis using the LunaScript RT SuperMix Kit (New England Biolabs) in a 20 μL reaction. cDNA synthesis was carried out at 55 °C for 10 min. PCR was then performed from 1.5 μL of cDNA with GoTaq G2 Colorless Master Mix (Promega) in a 25 μL reaction, using primers Ex-m50F (5′-AGGAAGTTAGAAGATCTGAGG-3′) and Ex-m55R (5′-GGAACTGCTGCAGTAATCTATGA-3′) under the following cycling conditions: 32 cycles of 95 °C for 30 s, 58 °C for 1 min, and 72 °C for 1 min. PCR products were electrophoresed on 1.5% agarose gels and quantified with ImageLab Software (Bio-Rad). Exon 51 skipping was also quantified by TaqMan real-time PCR as previously described,^70^ using specific assays targeting the exon 50–51 junction (assay Mm.PT.58.41685801; forward primer: 5′-CAAAGCAGCCTGACCGT-3′; reverse primer: 5′-TGACAGTTTCCTTAGTAACCACAG-3′; probe: 5′-TGGACTGAGCACTACTGGAGCCT-3′) and the exon 50–53 junction (forward primer: 5′-GCACTACTGGAGCCTTTGAA-3′; reverse primer: 5′-TTCCAGCCATTGTGTTGAATC-3′; probe: 5′-ACAGCTGCAGAACAGGAGACAACA-3′) (Integrated DNA Technologies). For each reaction, 150 ng of cDNA was used, and all measurements were performed in triplicate. qPCR was conducted under fast cycling conditions using a Bio-Rad CFX384 Touch Real-Time PCR Detection System. Data analysis was based on the absolute quantification method: copy numbers of the exon-skipped (exon 50–53) and unskipped (exon 50–51) products were determined using gBlock standards Ex49–54Δ52 and Ex49–54Δ51 + 52 (Integrated DNA Technologies). Exon 51 skipping levels were expressed as the percentage of total dystrophin transcripts, calculated as the ratio of skipped copies to the sum of skipped and unskipped copies.

Droplet digital PCR (ddPCR) was performed on the same RNA samples used for RT-PCR and qPCR analyses to quantify the expression of inflammatory markers. After reverse transcription, cDNA was subjected to ddPCR using the QX200 Droplet Digital PCR system (Bio-Rad), according to the manufacturer’s instructions. Specific assays were used to detect the following transcripts: TREM2 (assay Mm.PT.58.7992121), AIF1 (forward primer: 5′-CCCACCGTGTGACATCCA-3′; reverse primer: 5′-TGGTCCCCCAGCCAAGA-3′; probe: 5′-AGCTATCTCCGAGCTGCCCTGATTGG-3′), LAPTM5 (assay Mm.PT.58.31533533), TNF-α (forward primer: 5′-TGGGAGTAGACAAGGTACAACCC-3′; reverse primer: 5′-CATCTTCTCAAAATTCGAGTGACAA-3′; probe: 5′-CACGTCGTAGCAAACCACCAAGTGGA-3′), TGF-β1 (assay Mm.PT.58.11254750), CXCL10 (assay Mm.PT.58.43575827) (Integrated DNA Technologies), CDKN1A (assay Mm01303209_m1) and IL6 (assay Mm00446190_m1) (Thermo Fisher Scientific).

Each ddPCR reaction was prepared in a final volume of 40 μL containing 50 ng of cDNA, ddPCR Supermix for Probes (Bio-Rad), and the corresponding primer/probe assay. Droplets were generated with the QX200 Droplet Generator (Bio-Rad), and PCR amplification was performed on a C1000 Touch Thermal Cycler (Bio-Rad) under the following conditions: 95 °C for 10 min, 39 cycles of 94 °C for 30 s and 55 °C for 1 min, and a final step at 98 °C for 10 min. Droplets were then read on a QX200 Droplet Reader (Bio-Rad), and absolute transcript quantification was obtained using QuantaSoft Analysis Pro software (Bio-Rad). Results were expressed as copies per μL of input RNA. No-template controls were included in each run to monitor potential contamination.

### Quantification and statistical analysis

#### Statistics

Statistical analyses were conducted using R v4.3.2,^66^ Python v3.10,^67^ and GraphPad Prism v10.3.3. All paired-group comparisons, except for cell type proportion comparisons were performed using the two-sided Welch’s *t* test at the sample level with Benjamini-Hochberg correction. For ddPCR results, data normality was assessed prior to statistical testing; a one-way ANOVA test was applied when assumptions were met, whereas a non-parametric Kruskal–Wallis test was used. A significance threshold of α = 0.05 was used throughout; statistical significance was defined as ∗*p* < 0.05; ∗∗*p* < 0.01; ∗∗∗*p* < 0.001.

## Supplemental information

Document S1. Figures S1–S19

Table S1. List of genes and Dmd-related probes in the Xenium panel, related to Figure 1

Table S2. Marker gene list for cell type annotation in Xenium data, related to Figure 1

Table S3. Marker gene list for regional annotation in Xenium data, related to Figure 1

Table S4. Summary of ddPCR Assays and Reagents, related to STAR Methods
