# Supplementary material for: Evaluating exon skipping in the central nervous system in Duchenne muscular dystrophy using spatial transcriptomics
Source: iScience. 2026 Jul 23;29(8):116906. doi: 10.1016/j.isci.2026.116906 (PMC13427662; doi:10.1016/j.isci.2026.116906)
Supplement: Document S1. Figures S1–S19 [file mmc1.pdf]

**iScience, Volume *29***

## Supplemental information

# Evaluating exon skipping in the central nervous system in Duchenne muscular dystrophy using spatial transcriptomics

**Qirong Mao (毛启荣), Alireza Ahmadi, Sharon de Vries, Laura G.M. Heezen, Ophélie Vacca, Mathilde Doisy, Annemieke Aartsma-Rus, Maaike van Putten, Aurélie Goyenvalle, Ahmed Mahfouz, and Pietro Spitali**

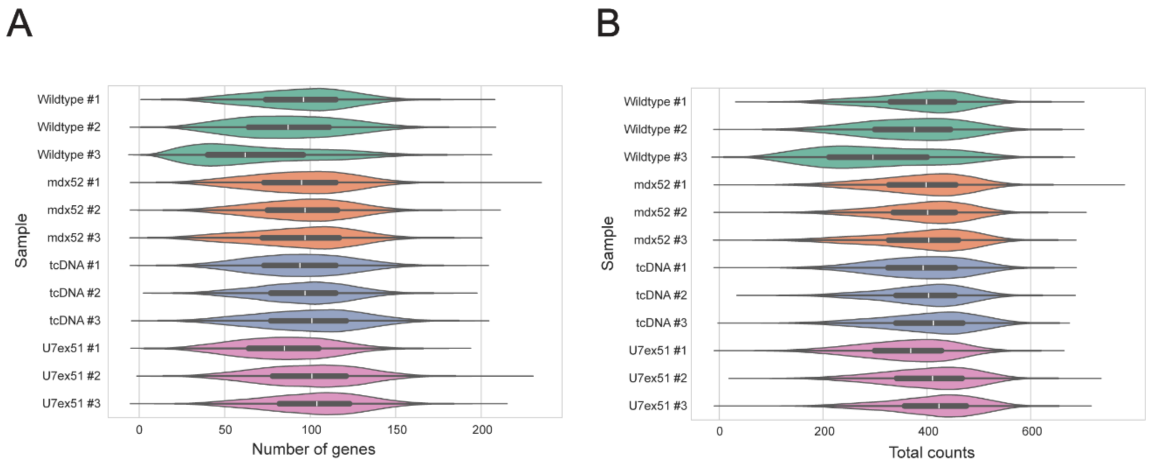


**Fig. S1. Quality view of Xenium data.**

Related to Figure 1

**(A–B)** Violin plots showing **(A)** the number of genes detected per cell and **(B)** the total counts per cell across samples and experimental groups.

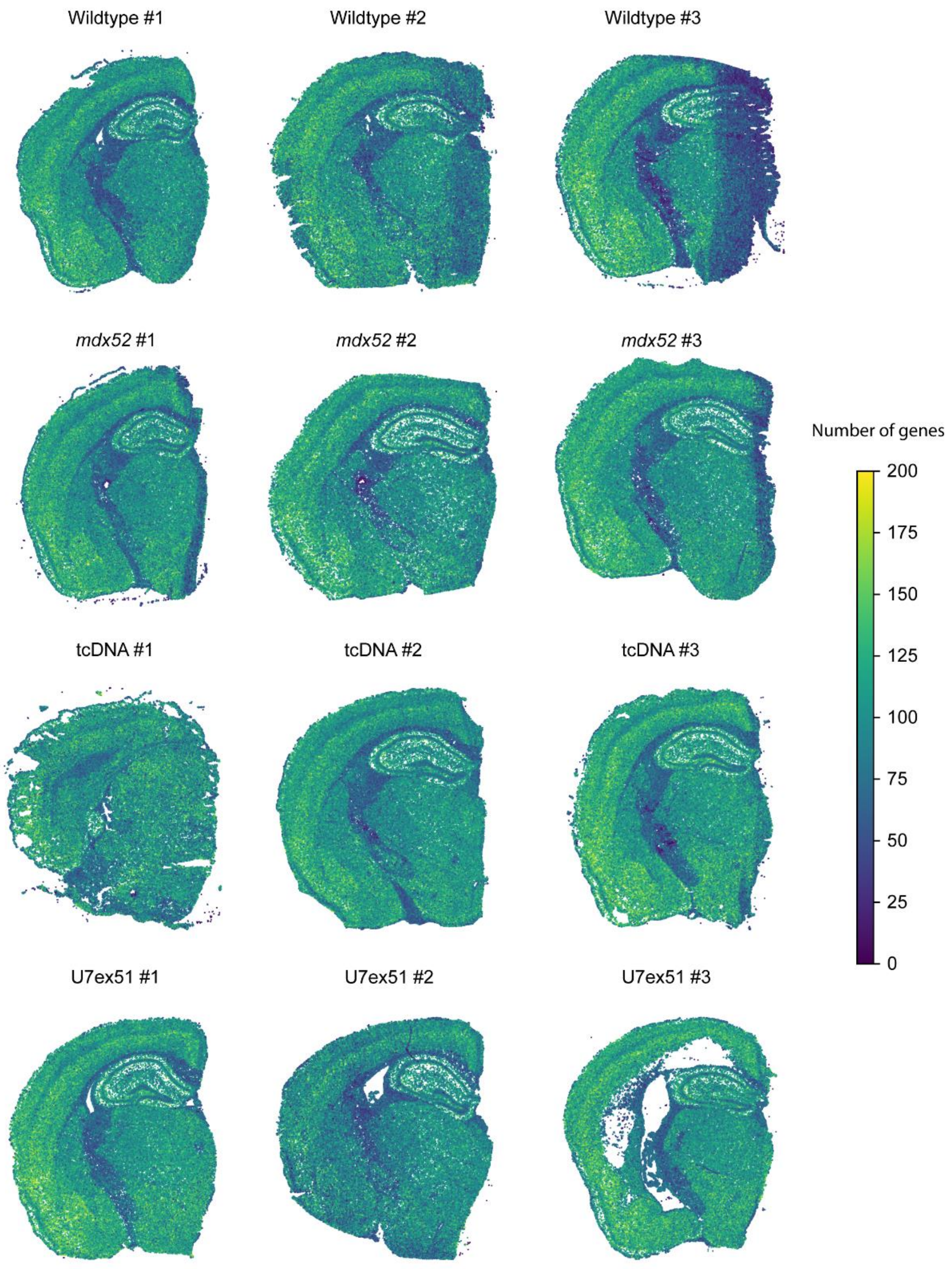


**Fig. S2. Spatial distribution of number of genes across samples**

Related to Figure 1

Spatial plots showing the distribution of number of genes distribution across samples

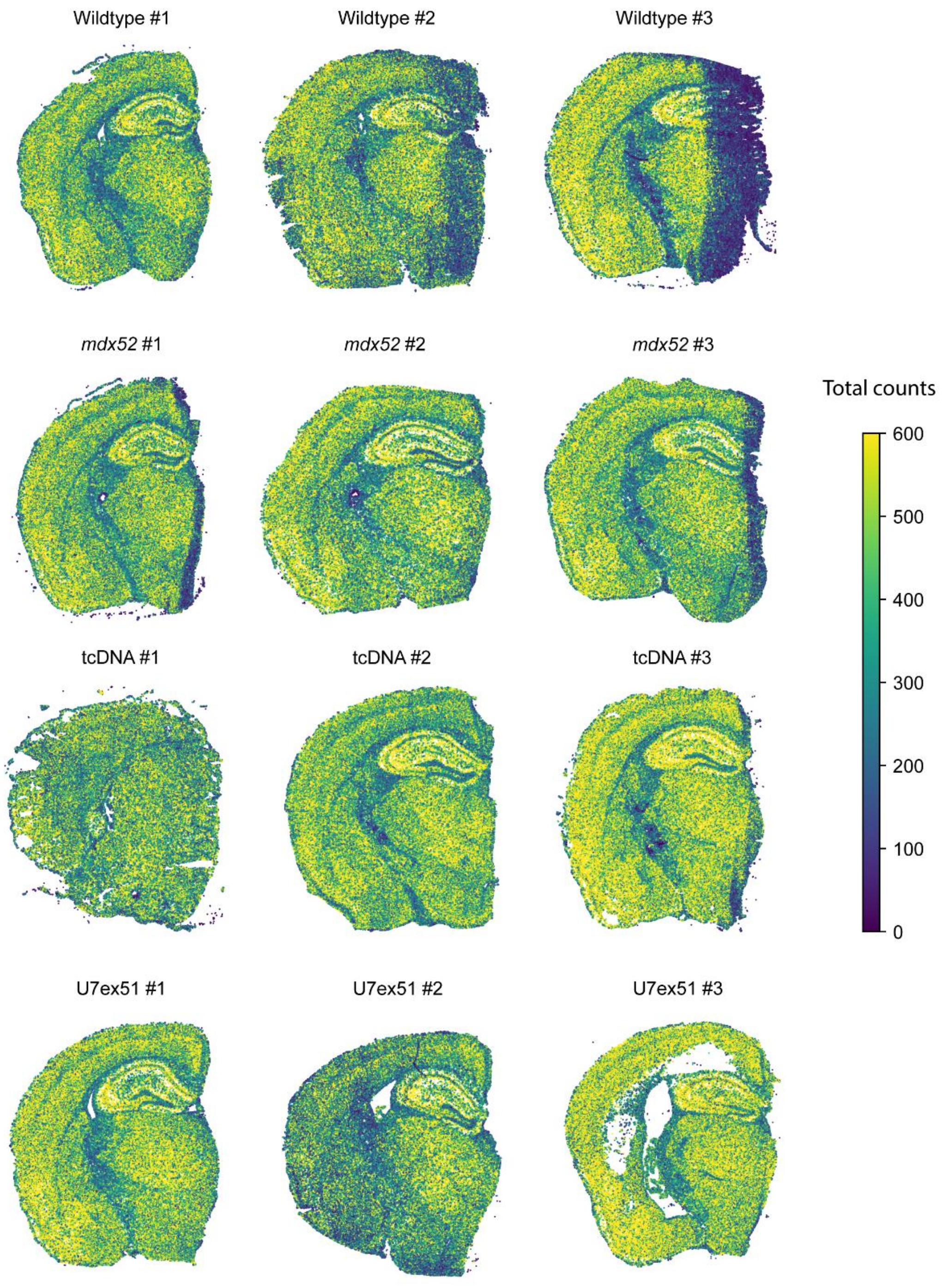


**Fig. S3. Spatial distribution of total counts across samples**

Related to Figure 1

Spatial plots showing the distribution of total counts distribution across samples

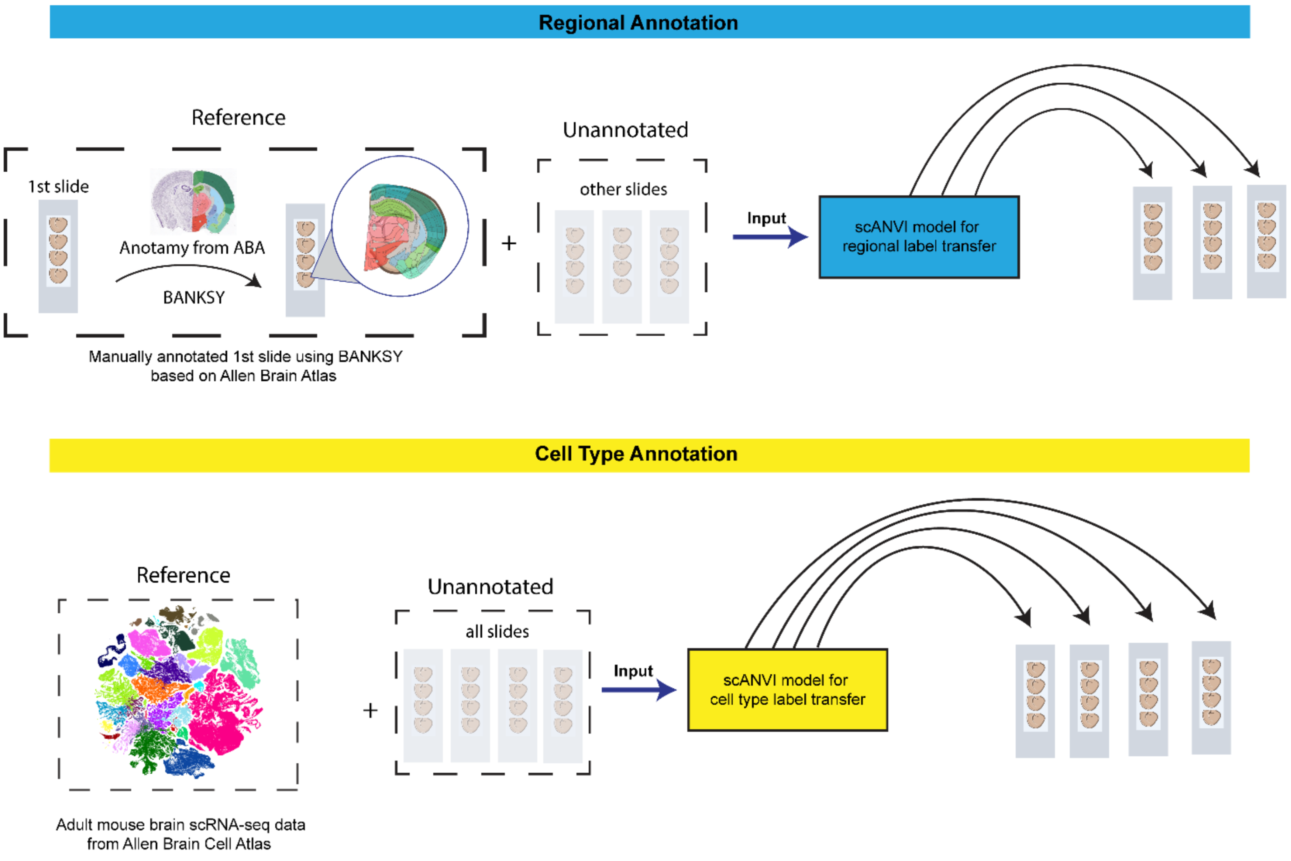


**Fig. S4. Schematic diagram of Xenium data annotation pipeline**

Related to Figure 1

Schematic diagram of Xenium data annotation:

(**Top**) Regional annotation: the first Xenium slide was annotated using BANKSY, guided by anatomical structures from the Allen Brain Atlas. This annotated slide, together with the rest of unannotated slides, was used as input to the scANVI model for label transfer.

(**Bottom**) Cell type annotation: A well-annotated, downsampled scRNA-seq atlas of the adult mouse brain from the Allen Brain Atlas was used as a reference, with all unannotated slides used as input to the scANVI model for cell type label transfer.

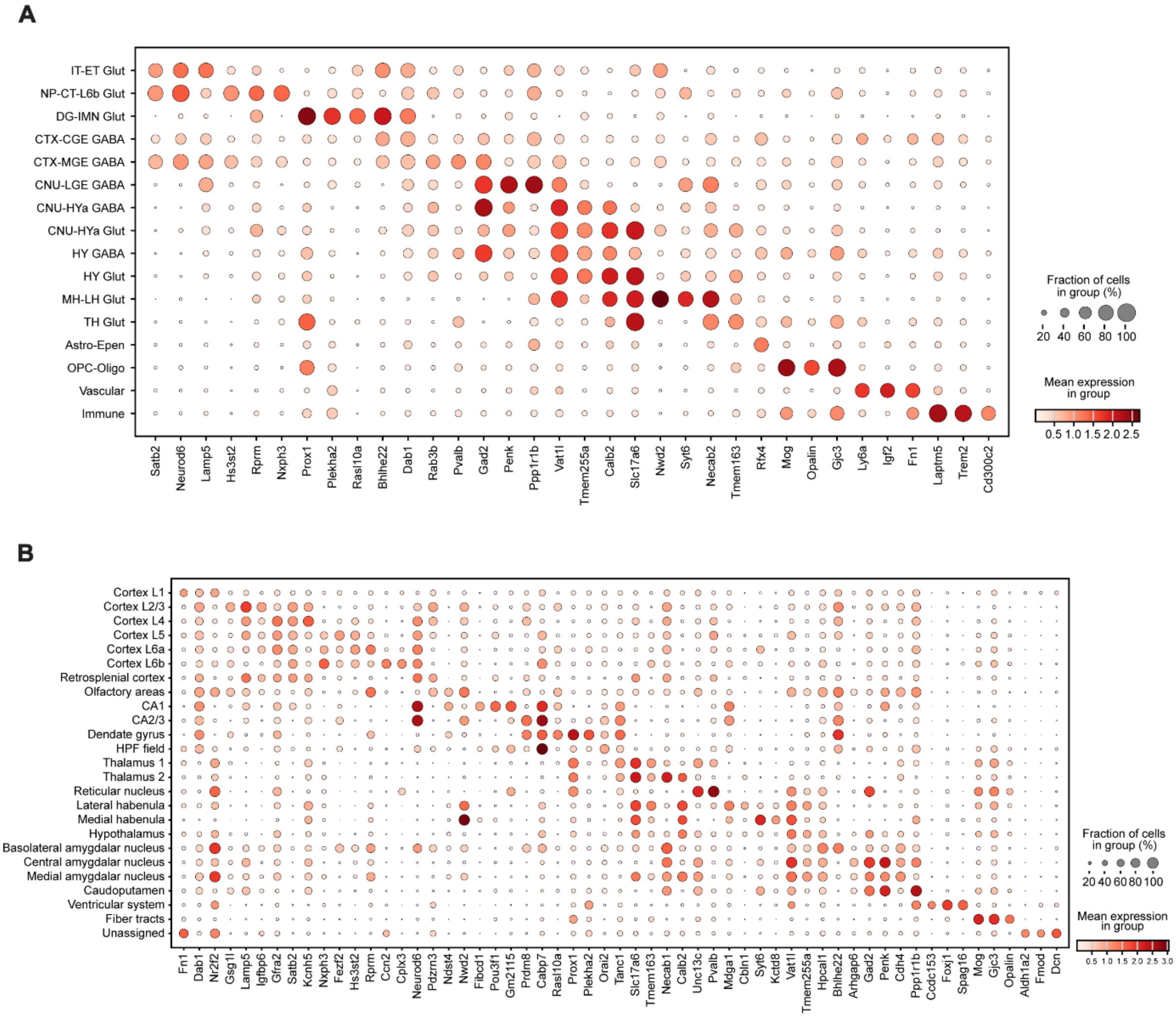


**Fig. S5. Top marker genes of annotated clusters for all samples**

Related to Figure 1

**(A–B)** Dot plots showing the mean expression of the top three marker genes for each annotated **(A)** cell type and **(B)** brain region group.

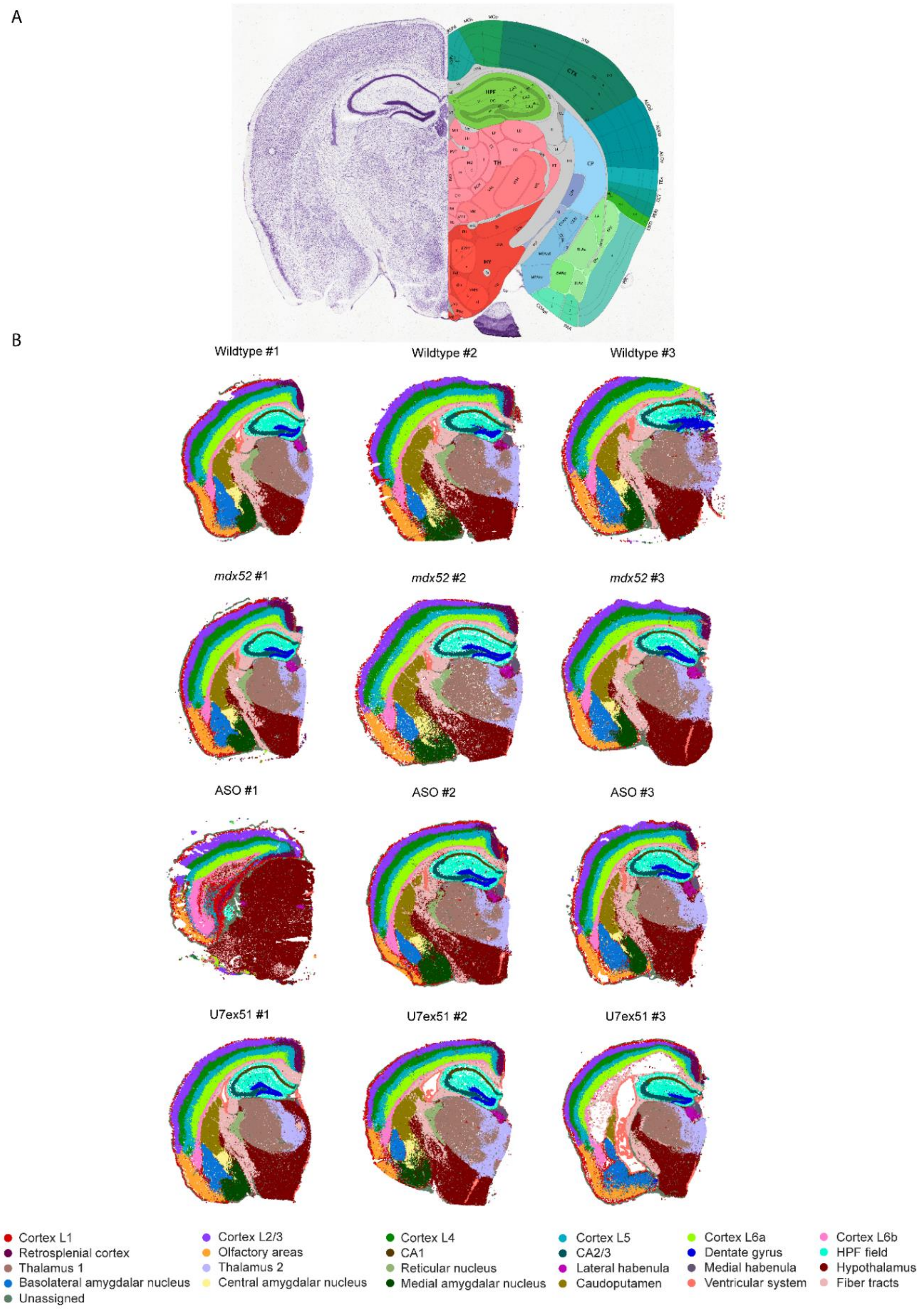


**Fig. S6. Regional annotation for all samples**

Related to Figure 1

(**A**) Reference anatomical annotation of P56 mouse brain from the Allen Brain Atlas

(**B**) Spatial plot of regional annotation across samples

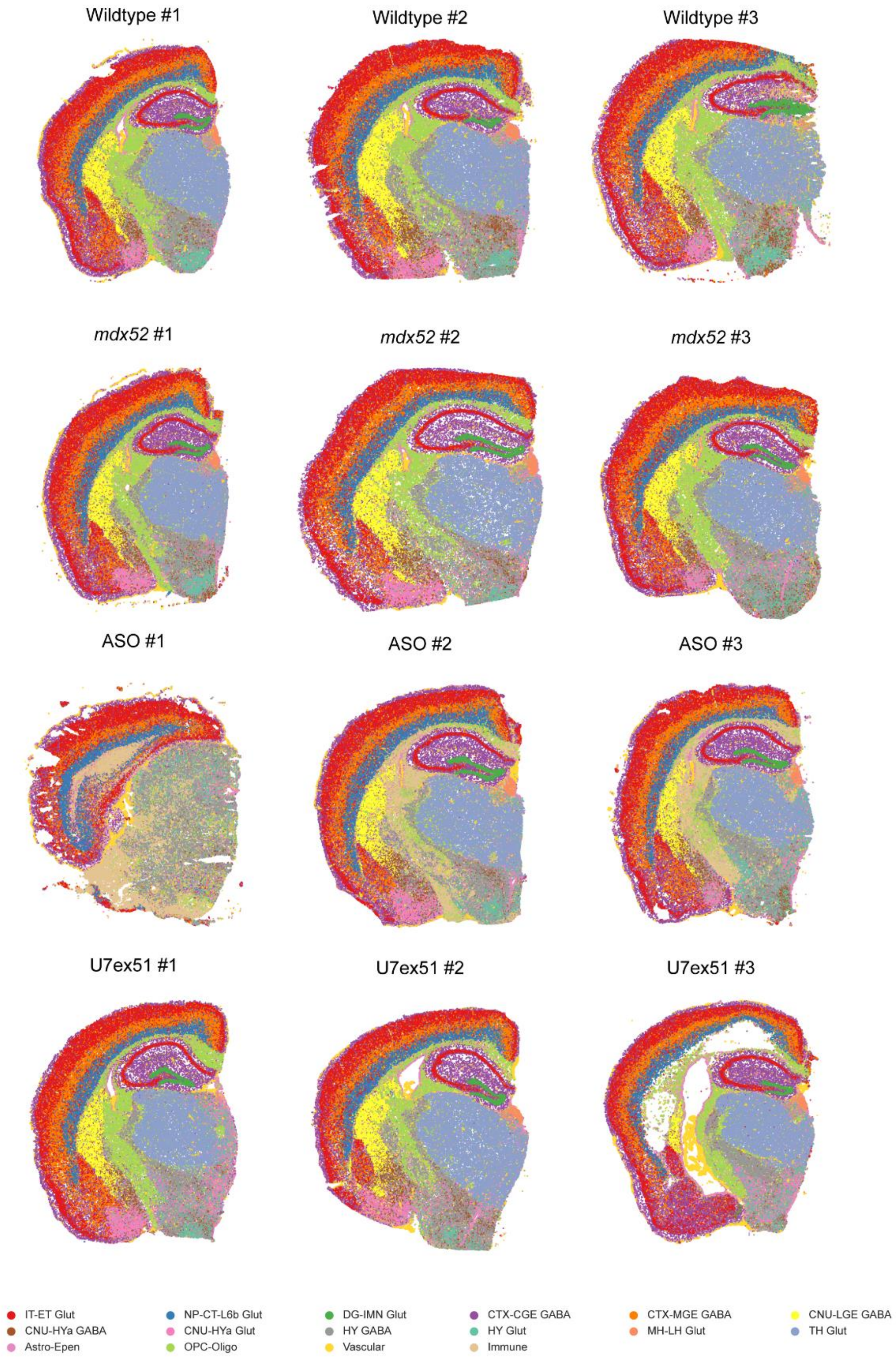


**Fig. S7. Cell type annotation for all samples**

Related to Figure 1

Spatial plots showing the distribution of cell type annotation across samples

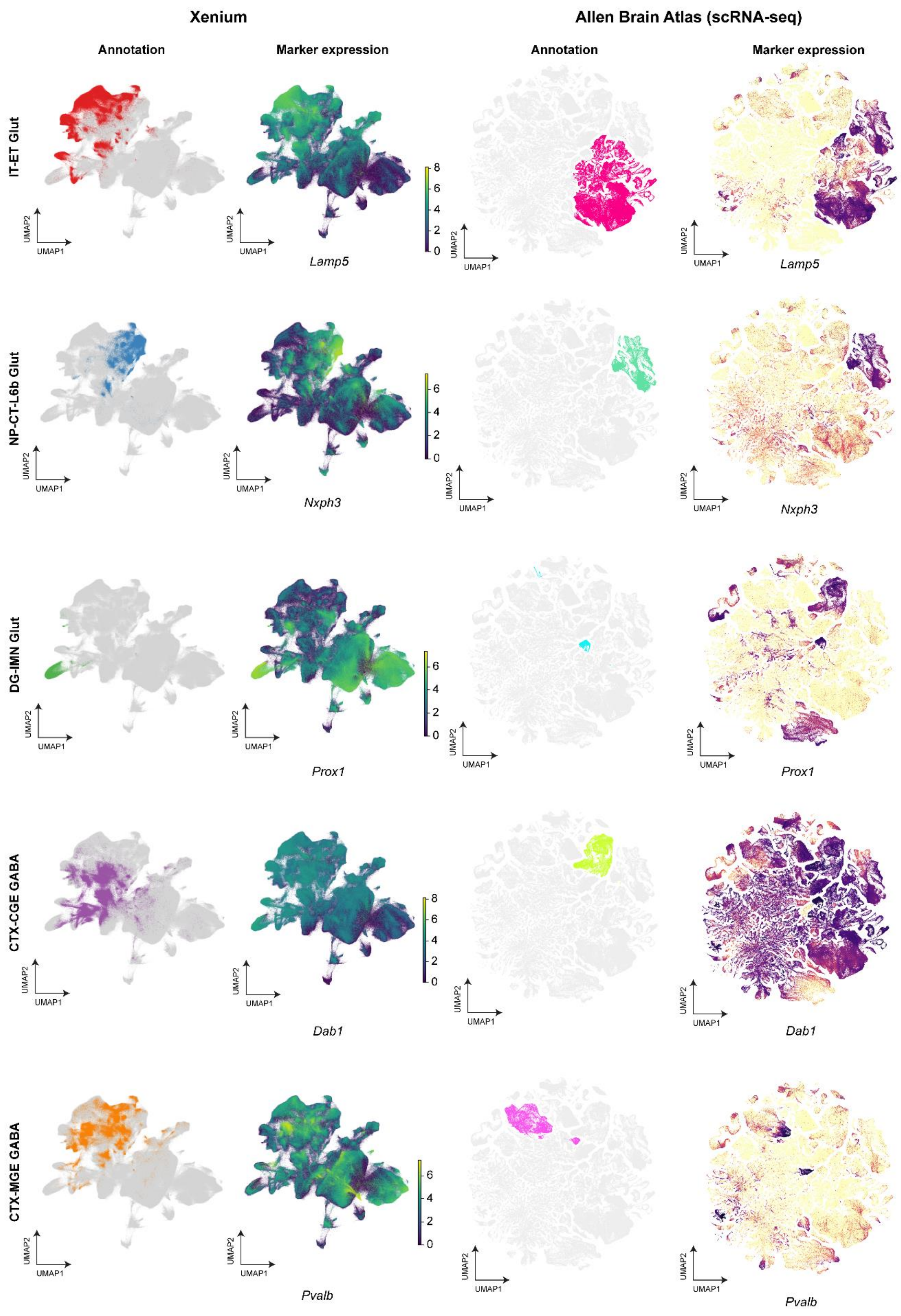
Xenium
Allen Brain Atlas (scRNA-seq)
Annotation
Marker expression
Annotation
Marker expression
IT-ET Glut
Lamp5
Lamp5
NP-CT-L6b Glut
Nxph3
Nxph3
DG-IMN Glut
Prox1
Prox1
CTX-CGE GABA
Dab1
Dab1
CTX-MGE GABA
Pvalb
Pvalb
UMAP1
UMAP2

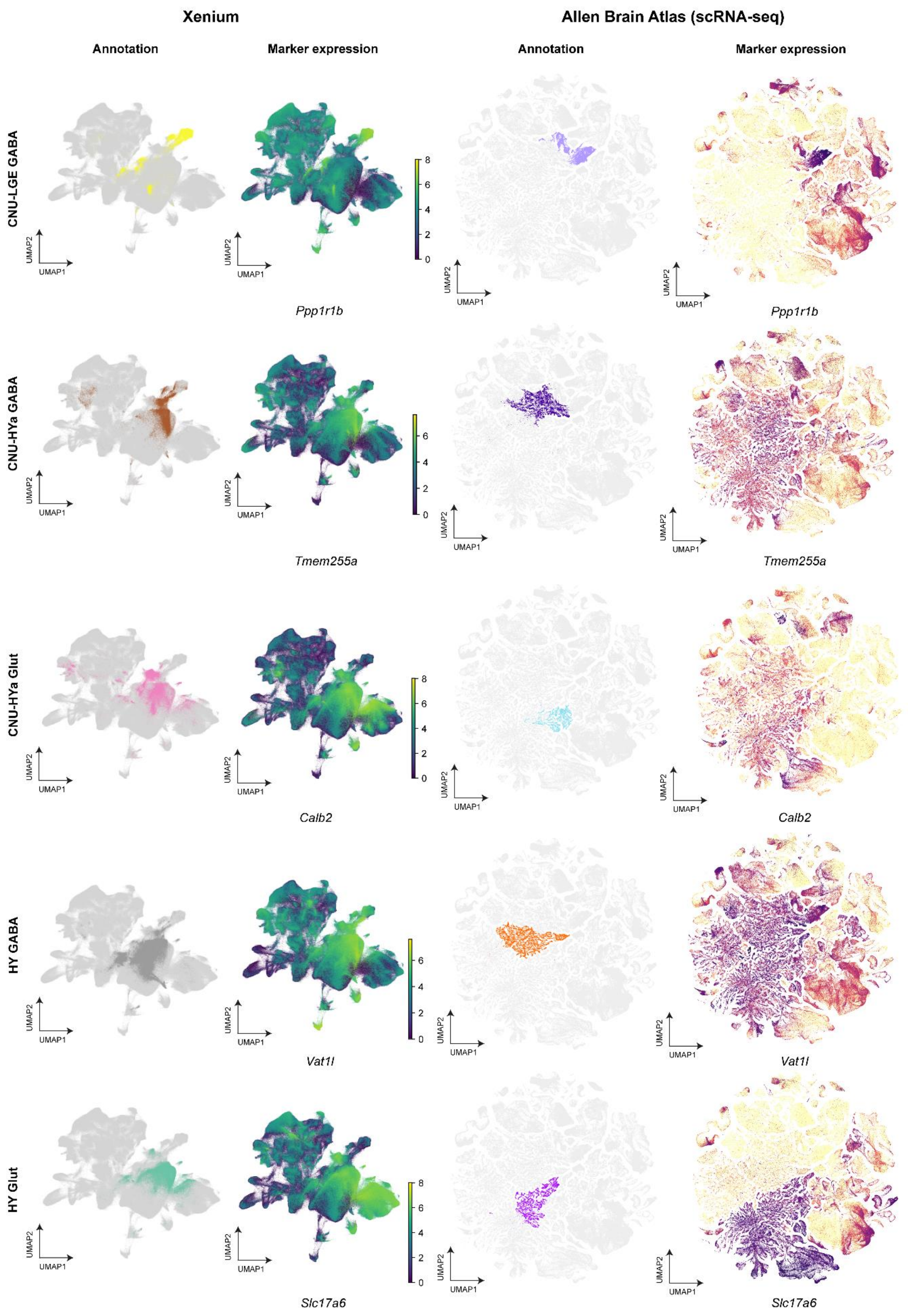
Xenium
Allen Brain Atlas (scRNA-seq)
Annotation
Marker expression
Annotation
Marker expression
CNU-LGE GABA
CNU-HYa GABA
CNU-HYa Glut
HY GABA
HY Glut
UMAP1
UMAP2
Ppp1r1b
Tmem255a
Calb2
Vat1l
Slc17a6

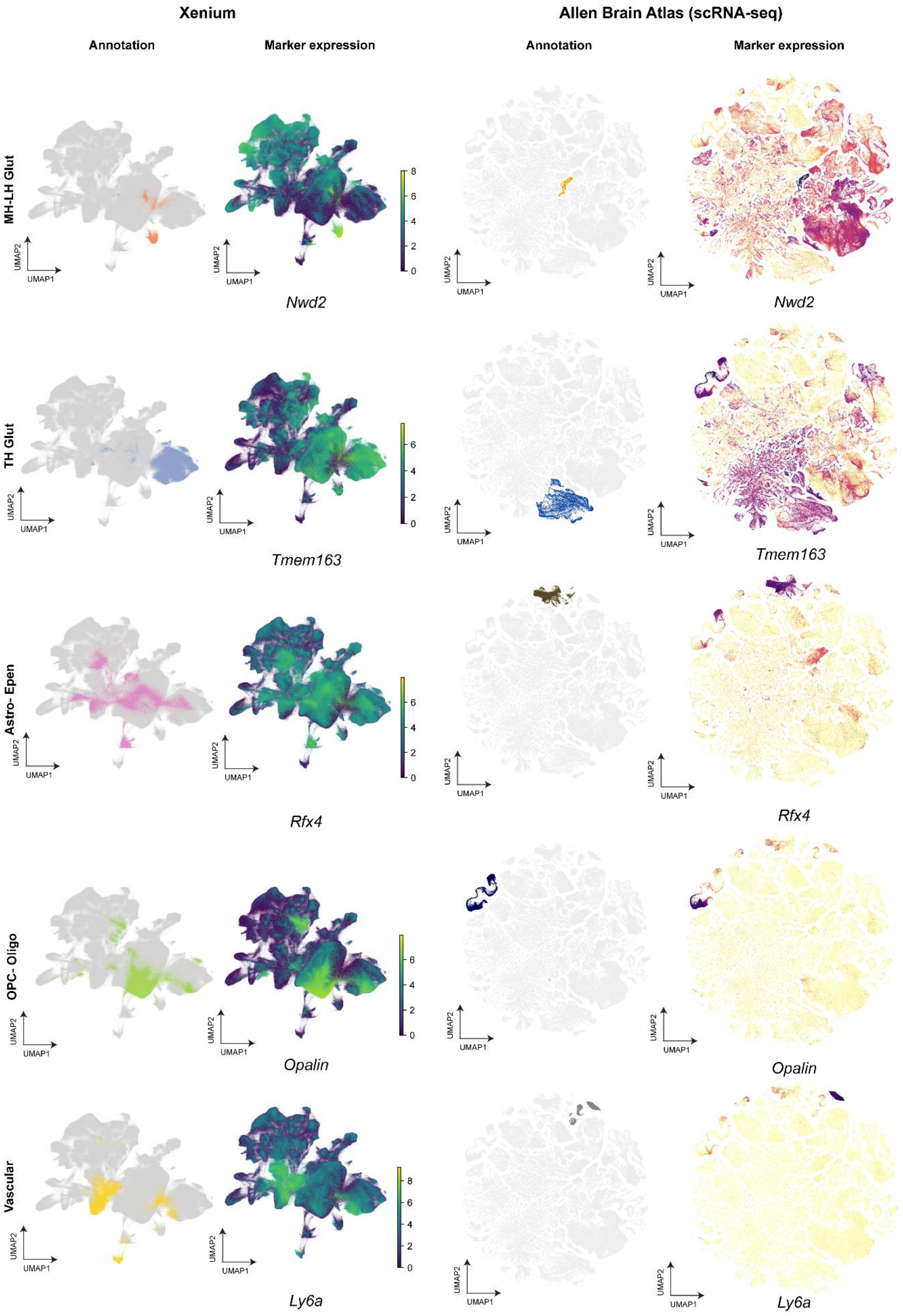
Xenium
Allen Brain Atlas (scRNA-seq)
Annotation
Marker expression
MH-LH Glut
TH Glut
Astro- Epen
OPC- Oligo
Vascular
UMAP1
UMAP2
Nwd2
Tmem163
Rfx4
Opalin
Ly6a

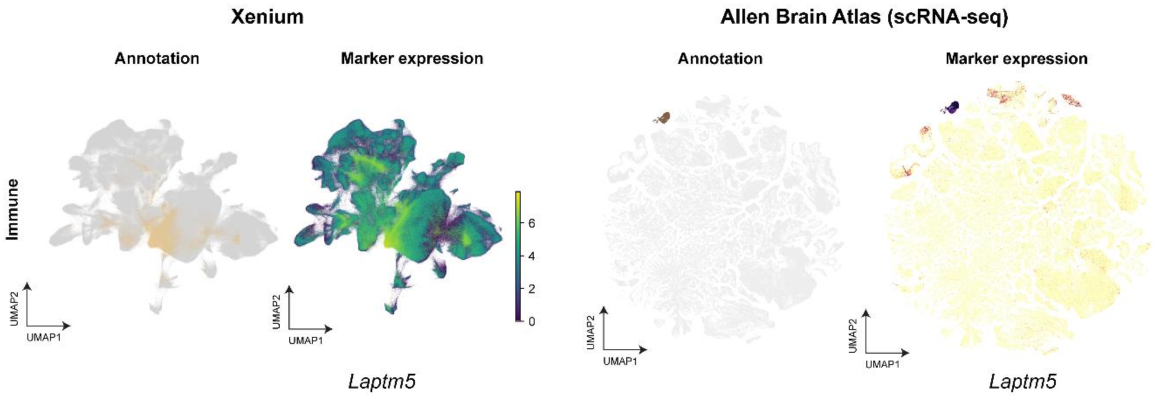


**Fig. S8-S11: Cross-platform comparison of cell-type annotation and marker expression between Xenium and reference scRNA-seq data**

Related to Figure 1

**(Left)** UMAP of the merged Xenium dataset, showing cell-type annotations for all cells and the log-normalized expression of representative marker genes.

**(Right)** UMAP of the reference single-cell RNA-seq dataset from the Allen Brain Atlas, showing the corresponding cell-type annotations and the log-normalized expression of the same marker genes.

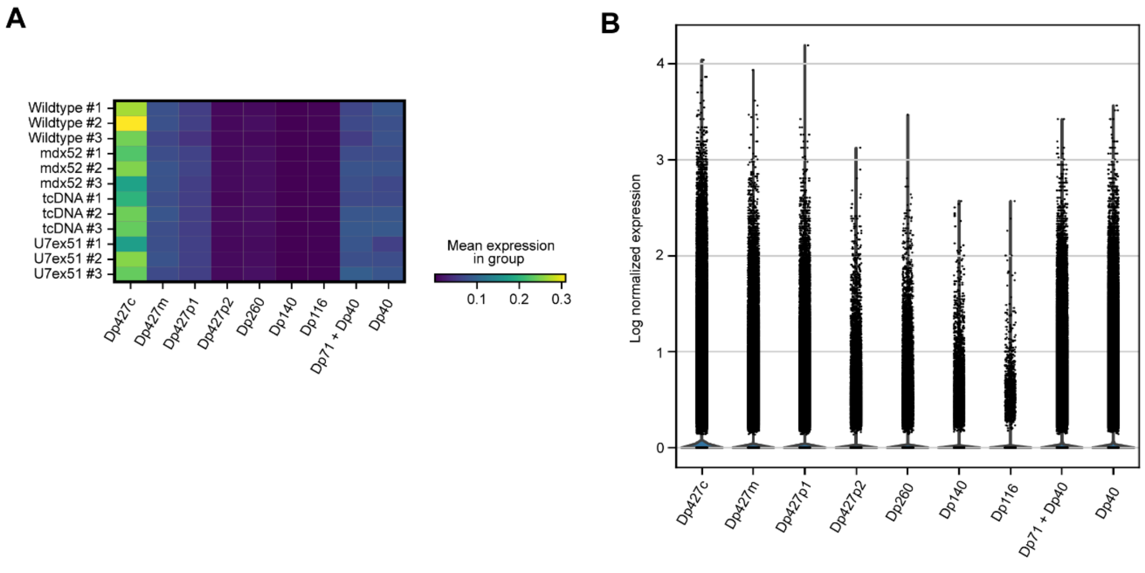


**Fig. S12. Quality of Xenium custom probes targeting *Dmd* isoforms**

Related to Figure 1

**(A)** Heatmap showing log-normalized mean expression of *Dmd* isoform probes across samples targeted by Xenium custom probes.

**(B)** Violin plot illustrating overall *Dmd* isoform expression across all samples; each dot represents expression in an individual cell.

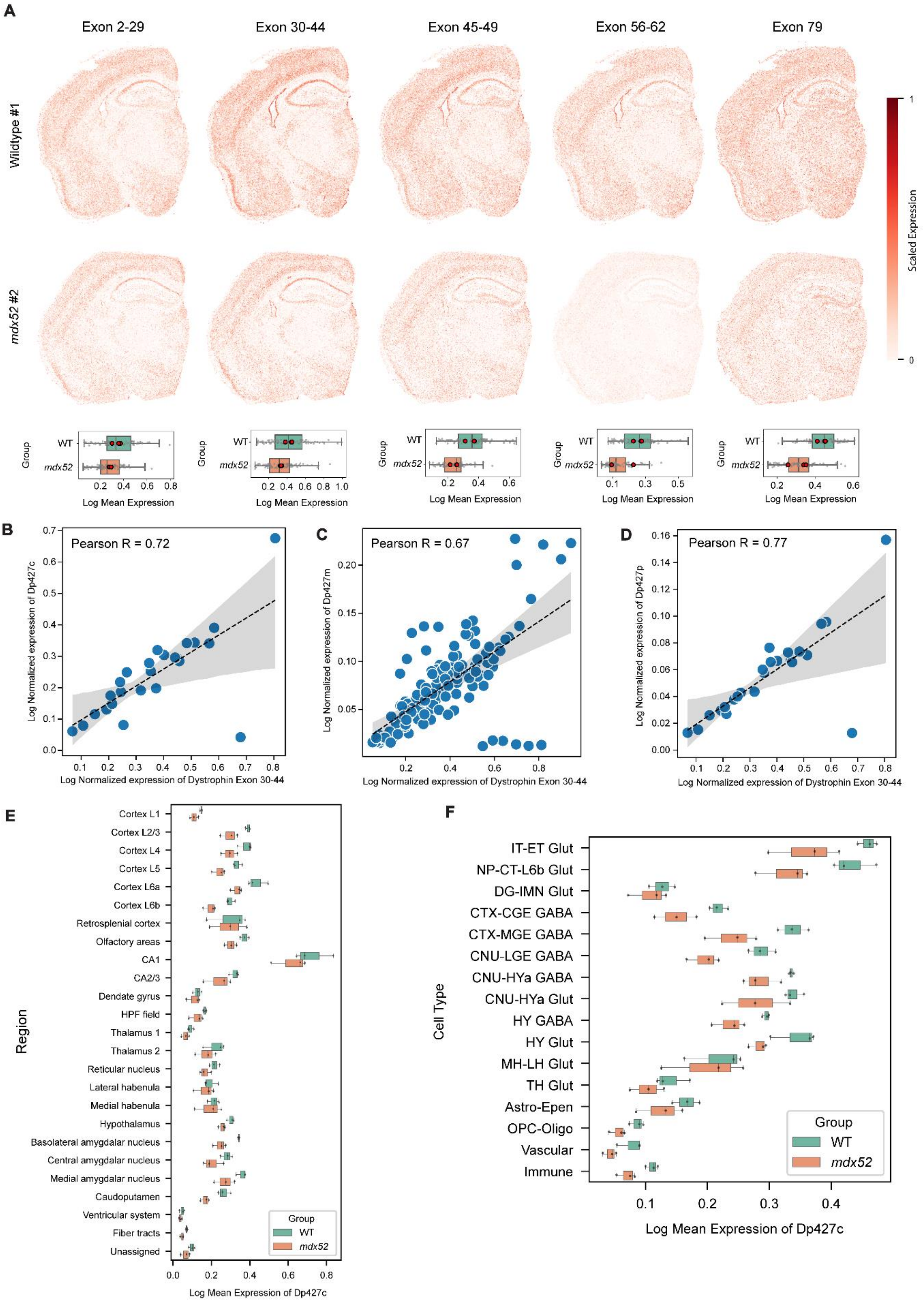
A
Exon 2-29
Exon 30-44
Exon 45-49
Exon 56-62
Exon 79
Wildtype #1
mdx52 #2
Scaled Expression
1
0
Group
WT
mdx52
Log Mean Expression
B
Pearson R = 0.72
Log Normalized expression of Dp427c
Log Normalized expression of Dystrophin Exon 30-44
C
Pearson R = 0.67
Log Normalized expression of Dp427m
Log Normalized expression of Dystrophin Exon 30-44
D
Pearson R = 0.77
Log Normalized expression of Dp427p
Log Normalized expression of Dystrophin Exon 30-44
E
Region
Cortex L1
Cortex L2/3
Cortex L4
Cortex L5
Cortex L6a
Cortex L6b
Retrosplenial cortex
Olfactory areas
CA1
CA2/3
Dendate gyrus
HPF field
Thalamus 1
Thalamus 2
Reticular nucleus
Lateral habenula
Medial habenula
Hypothalamus
Basolateral amygdalar nucleus
Central amygdalar nucleus
Medial amygdalar nucleus
Caudoputamen
Ventricular system
Fiber tracts
Unassigned
Group
WT
mdx52
Log Mean Expression of Dp427c
F
Cell Type
IT-ET Glut
NP-CT-L6b Glut
DG-IMN Glut
CTX-CGE GABA
CTX-MGE GABA
CNU-LGE GABA
CNU-HYa GABA
CNU-HYa Glut
HY GABA
HY Glut
MH-LH Glut
TH Glut
Astro-Epen
OPC-Oligo
Vascular
Immune
Group
WT
mdx52
Log Mean Expression of Dp427c

**Fig. S13. Regional probe expression across wildtype and *mdx52* samples**

Related to Figure 2
**(A)** (Top) Spatial distribution of *Dmd* regional probes in WT and *mdx52* mouse brains colored by scaled expression of each *Dmd* regional probes. (Bottom) Boxplot with overlaid dot plots showing the log-transformed mean expression of each *Dmd* regional probes across samples and brain regions (black) and the log-transformed mean expression per mouse in WT and mdx52 groups (red). Statistical analysis was performed using Welch's t-test with Benjamini–Hochberg (BH) correction at the sample level.

**(B-D)** Dot plot showing the correlation between *Dmd* regional probe exon 30-44 and **(B)** Dp427c ; **(C)** Dp427m; **(D)** Dp427p expression (log-normalized) across brain regions in WT and *mdx52* samples. **(E-F)** Expression of Dp427c across brain regions and cell types in WT and *mdx52* mice. Statistical analysis by the Welch's t-test with BH correction. $*P < 0.05$

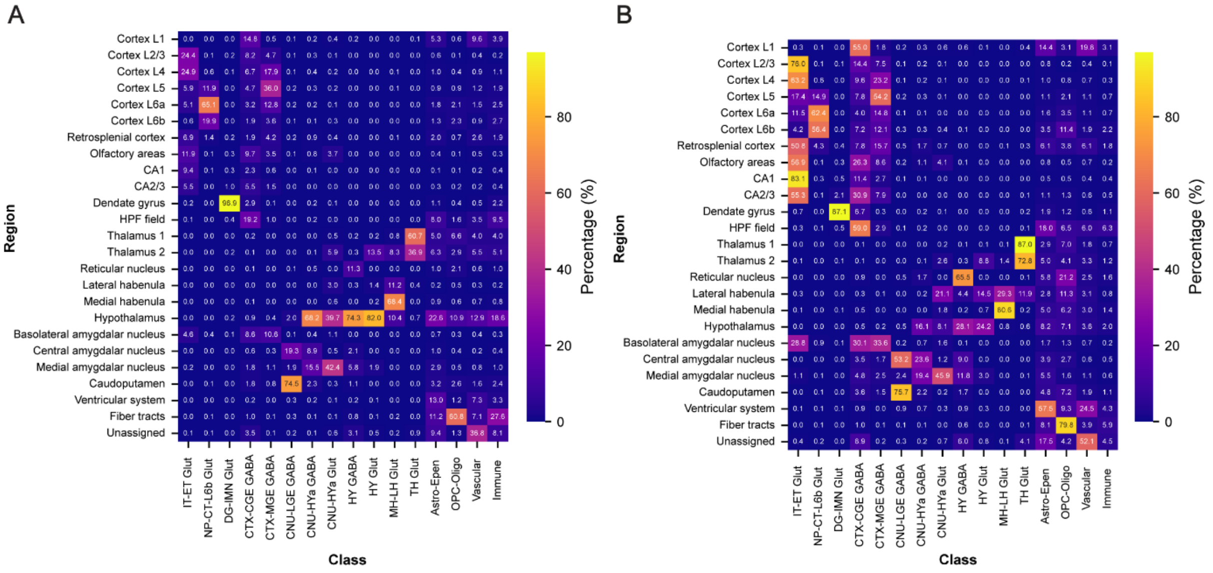


**Fig. S14. Cell type composition and distribution across regions**

Related to Figure 3

**(A-B)** Heatmap of **(A)** cell type composition per region and **(B)** distribution of cell types across regions

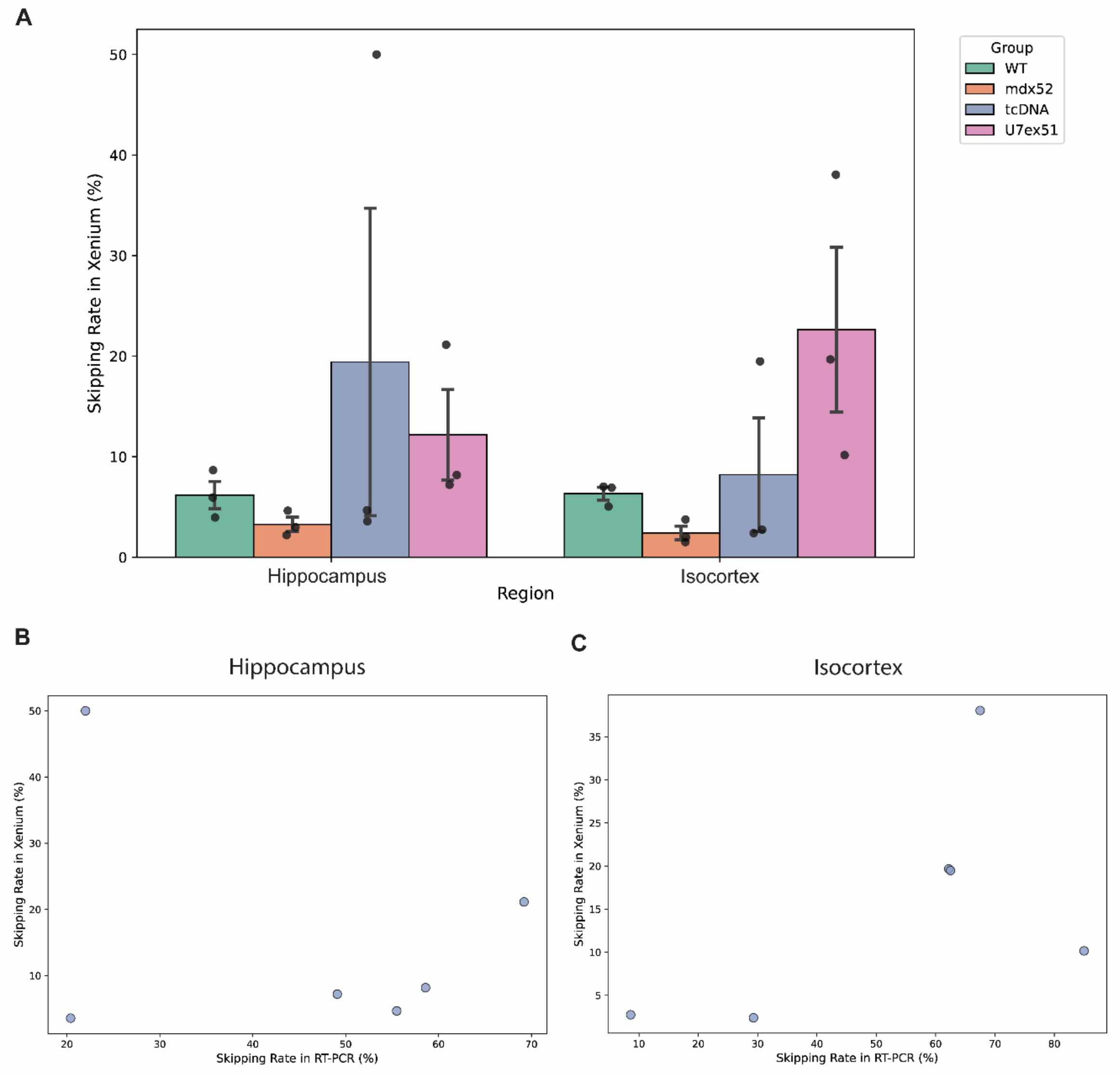


**Fig. S15. Comparison of exon-skipping rates measured by Xenium and RT-PCR in the hippocampus and isocortex.**

Related to Figure 4

**(A)** Exon-skipping rates calculated from Xenium data across the hippocampus and isocortex. **(B-C)** Scatter plots comparing exon-skipping rates measured by RT-PCR and Xenium for each sample in the **(B)** hippocampus and **(C)** isocortex.

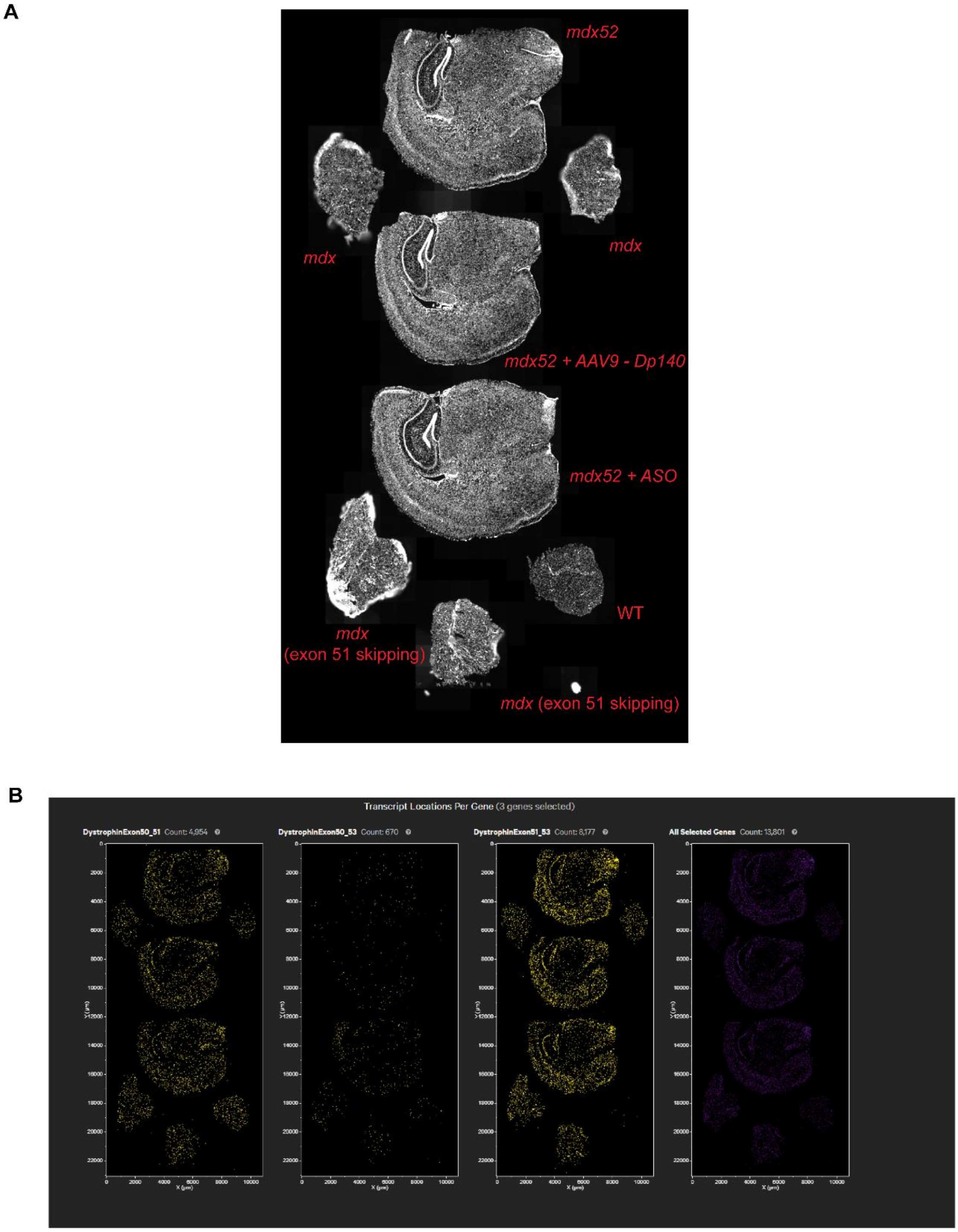


**Fig. S16. Quality assessment of exon junction probe validation across different tissues.**

Related to Figure 4

**(A)** Samples included on the Xenium slide comprised *mdx52* brain samples treated with PBS, mini-Dp140 dystrophin cDNA, or ASO, together with murine *mdx* muscle samples with or without exon 51 skipping

**(B)** Spatial scatter plots illustrating the distribution of exon junction probes across the samples.

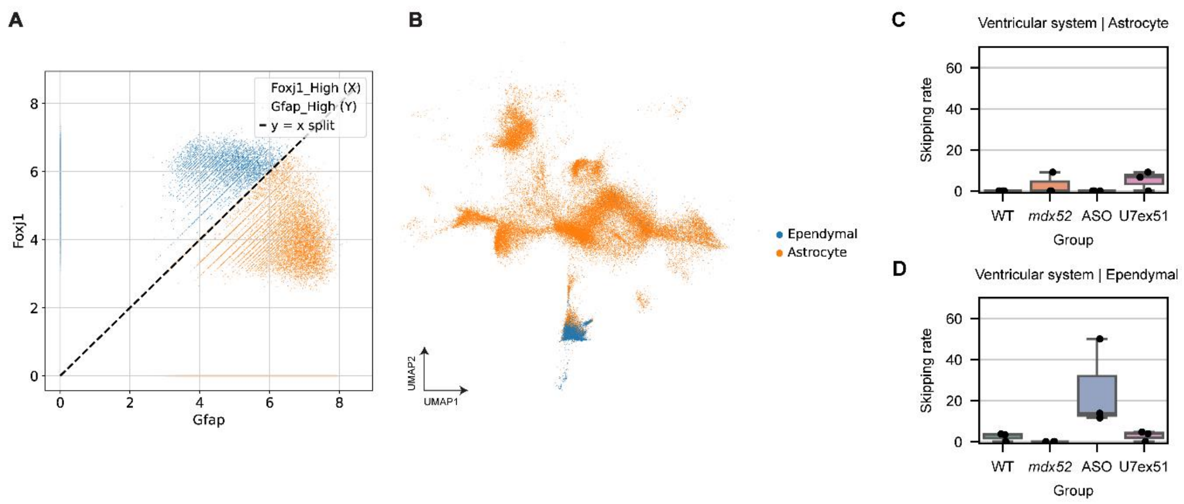


**Fig. S17. Reclustering of the Astro–Ependymal (Astro-Epen) cell population and skipping rate analysis.**

Related to Figure 4

(**A**) Scatter plot showing reclustering of Astro-Epen cells based on *Foxj1* and *Gfap* expression (log-normalized). Cells were separated using a $y = x$ threshold, delineating *Foxj1*-high (ependymal-like) and *Gfap*-high (astrocyte-like) populations.

(**B**) UMAP visualization of the reclustered Astro-Epen population, with cells annotated as ependymal or astrocyte based on marker gene expression.

(**C-D**) Quantification of skipping rates in (**C**) astrocytes and (**D**) ependymal within the ventricular system across groups

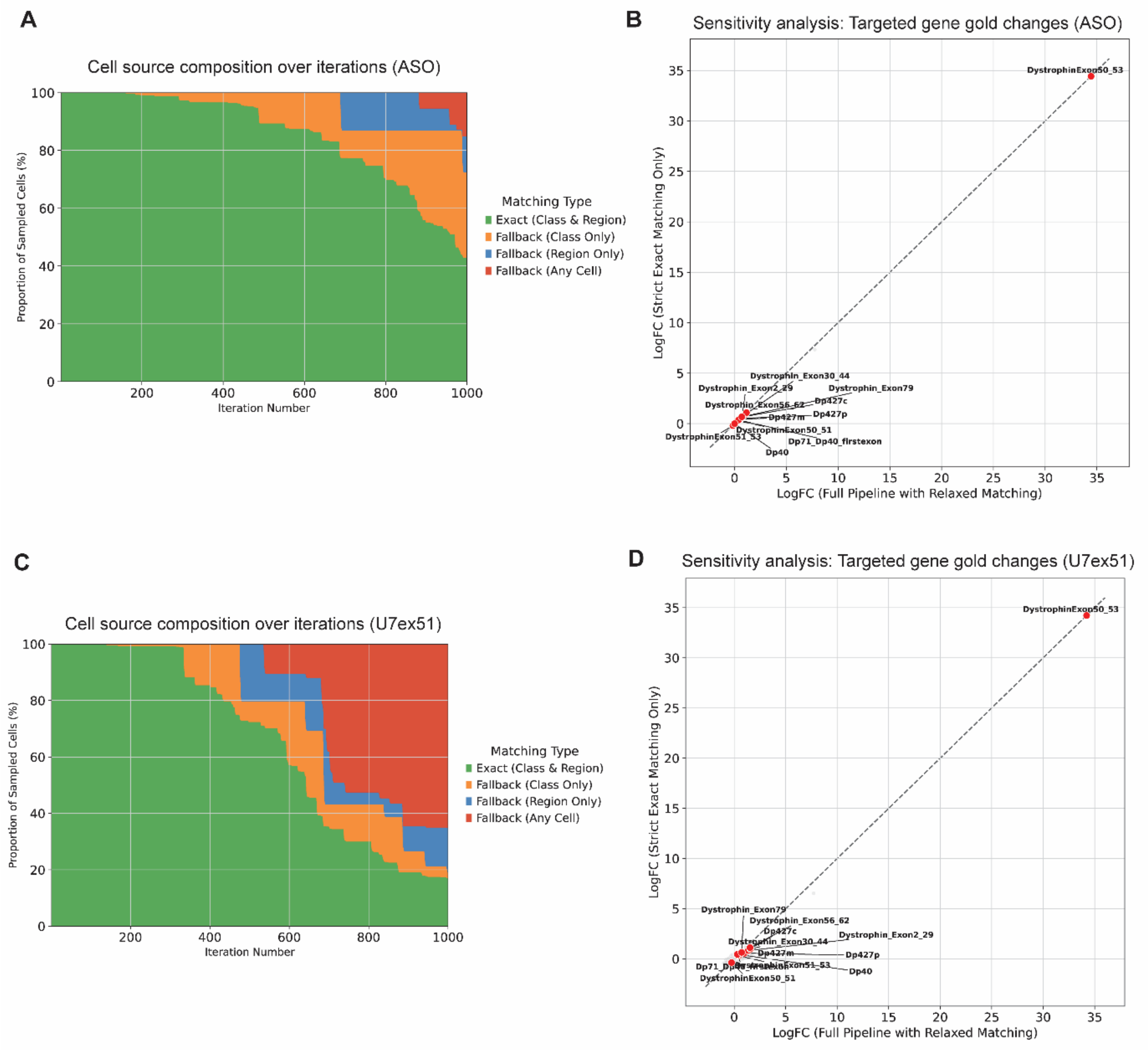


**Fig. S18. Sensitivity analysis of the permutation test in the ASO- and U7ex51-treated samples.**

Related to Figure 5

**(A, C)** Composition of matched source cells across 1,000 iterations for the **(A)** ASO- and **(C)** U7ex51-treated samples, categorized by matching type: exact match by class and region, followed by fallback matches by class, then by region, and finally matches to any cell.

**(B, D)** Scatter plots comparing log fold-change (LogFC) estimates obtained using the full pipeline with relaxed matching versus strict exact matching for targeted dystrophin-related genes in the **(B)** ASO and **(D)** U7ex51 datasets. The dashed diagonal indicates agreement between the two approaches. Red dots represent the *Dmd* related probes, grey dots represent the rest of the probes in the panel.

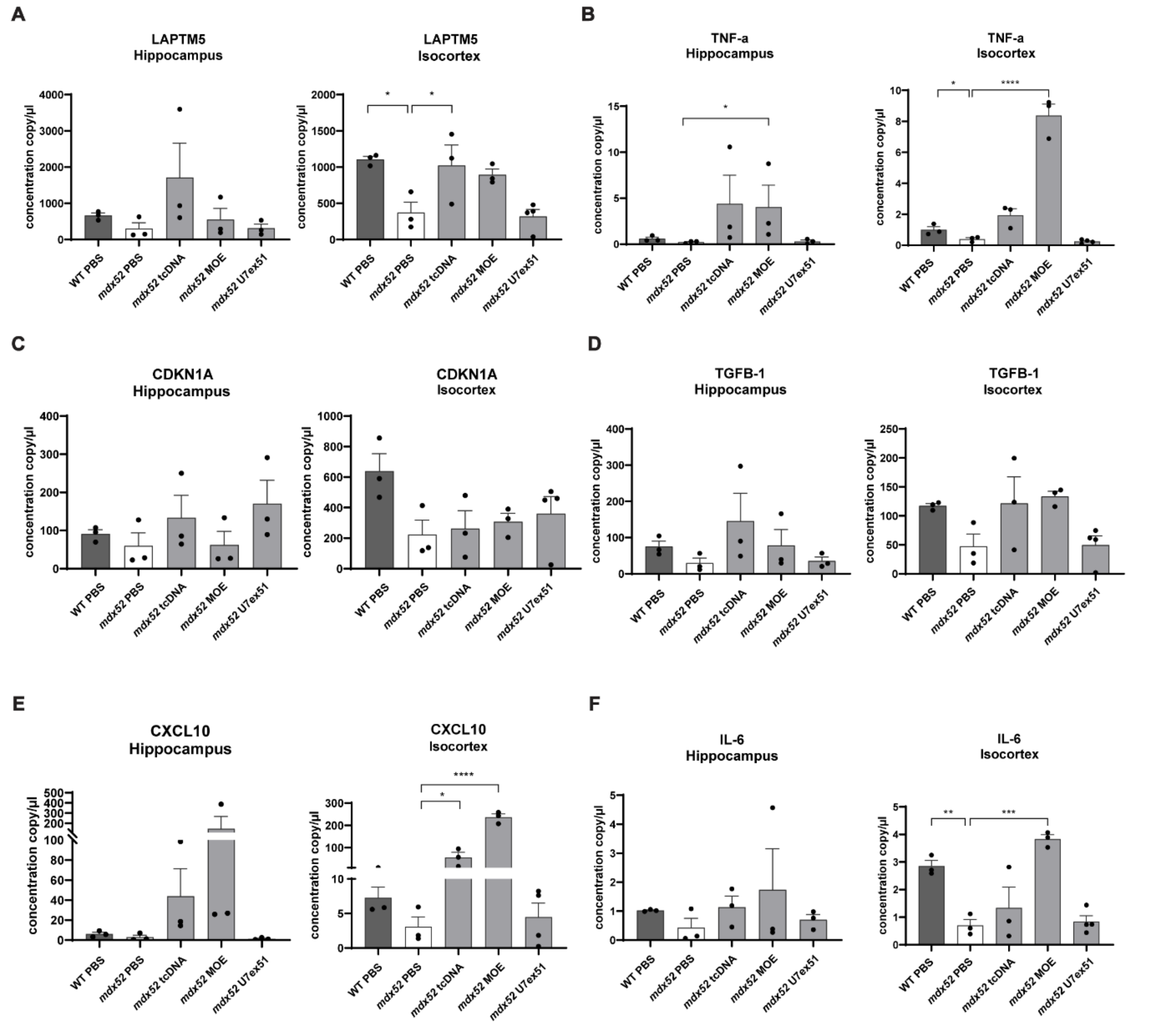


**Fig. S19. Quantification of immune marker expression in brains treated with ASOs of different chemical backbones**

Related to Figure 6

**(A–F)** Absolute quantification of mRNA levels measured by droplet digital PCR (ddPCR) in the hippocampus (left) and isocortex (right) for: **(A)** LAPTM5 (**p* = 0.0197 WT vs mdx52; **p* = 0.0379 *mdx52* PBS vs tcDNA51), **(B)** TNF-α (hippocampus **p* = 0.0412; isocortex **p* = 0.0472), **(C)** CDKN1a, **(D)** TGF-β1, **(E)** CXCL10 (**p* = 0.0283; *****p* < 0.0001), and **(F)** IL6 (**p* = 0.0067; ***p* = 0.0004). Experimental groups include wild type mice control (WT PBS), mdx52 mice control (*mdx52 PBS*), *mdx52* tcDNA51, *mdx52* MOE51, and *mdx52* AAV-U7-51. Results are expressed as copies per µL of input RNA and shown as mean ± SEM (n = 3–4 per group). Data normality was assessed prior to statistical testing; one-way ANOVA test was applied when assumptions were met, otherwise a non-parametric Kruskal–Wallis test was used. Statistical significance between groups is indicated by horizontal bars.
